# Integrated host–microbe biomarkers for early diagnostic classification of paediatric LRTI in mechanically ventilated children

**DOI:** 10.1186/s12931-026-03829-6

**Published:** 2026-07-27

**Authors:** Zongtai Wu, Gehad Youssef, Iain R. L. Kean, Zhenguang Zhang, John A. Clark, Nazima Pathan, Namshik Han

**Affiliations:** 1 Milner Therapeutics Institute, University of Cambridge, Cambridge, UK; 2 Department of Public Health and Primary Care, University of Cambridge, Cambridge, UK; 3 Cambridge Centre for AI in Medicine, University of Cambridge, Cambridge, UK; 4 Cambridge Stem Cell Institute, University of Cambridge, Cambridge, UK; 5 Department of Quantum Information, Institute for Convergence Science Academy, Yonsei University, Seoul, Republic of Korea; 6 Department of Paediatrics, University of Cambridge, Cambridge, UK; 7 Paediatric Intensive Care Unit, Addenbrooke’s Hospital, Cambridge, UK; 8 Department of Nano Biomedical Engineering (NanoBME), Advanced Science Institute, Yonsei University, Seoul, Republic of Korea; 9 Center for Nanomedicine, Institute for Basic Science (IBS), Seoul, Republic of Korea

**Keywords:** Lower respiratory tract infection, Paediatric intensive care, Respiratory syncytial virus, Haemophilus influenzae, Host transcriptomics, Metagenomic sequencing, Diagnostic biomarkers, Multi-omics integration

## Abstract

**Background:**

Early diagnosis of paediatric lower respiratory tract infection (LRTI) in intensive care is difficult because clinical features overlap with non-infectious respiratory failure and conventional microbiology has limited sensitivity. We aimed to integrate airway microbial and host-response signals to identify early diagnostic biomarkers in mechanically ventilated children.

**Methods:**

We re-analysed airway metagenomic sequencing and tracheal aspirate transcriptomics from 261 ventilated paediatric intensive care unit (PICU) patients using differential expression, protein–protein interaction network analysis, and machine-learning feature selection. Findings were evaluated in an exploratory assessment of biologically related cytokine markers within an independent prospective cohort (RASCALS; *n* = 100 enrolled, *n* = 78 analysed) using non-bronchoscopic mini-bronchoalveolar lavage (mini-BAL), plasma cytokines, and clinician-adjudicated diagnoses.

**Results:**

Respiratory syncytial virus and *Haemophilus influenzae* were the only pathogens consistently enriched in LRTI. Host transcriptomics showed activation of interferon, cytokine, and chemokine signalling. Network-guided feature selection identified a seven-gene panel (IRF7, FFAR3, GZMB, FABP4, FN1, CXCL5, BCAR1) with high diagnostic performance in cross-validation (median F1 0.94), comparable to a published 14-gene model. In RASCALS, a logistic model using mini-BAL IL-1β, IL-4, and IL-8 classified bacterial LRTI with 65% accuracy (sensitivity 70%, specificity 63%), while blood IL-6 and TRAIL achieved 82% accuracy (sensitivity 79%, specificity 83%).

**Conclusions:**

Integrating airway microbial profiles with host-response biomarkers supports earlier and more specific LRTI diagnostic classification in ventilated children. The seven-gene panel and cytokine combinations are candidates for rapid PCR- or immunoassay-based bedside tests to inform antimicrobial stewardship.

**Supplementary Information:**

The online version contains supplementary material available at https://doi.org/10.1186/s12931-026-03829-6.

## Background

Lower respiratory tract infection (LRTI) is a major cause of morbidity and mortality among children globally and remains a leading cause of death from infection in those under five years of age [1, 2]. Community-acquired pneumonia remains a major driver of paediatric hospitalisation and severe respiratory illness, and dysregulated innate and adaptive immune responses contribute to clinical heterogeneity in infection severity and recovery [3, 4]. In intensive care, these challenges contribute to diagnostic uncertainty and empirical antibiotic use, with downstream consequences for antimicrobial resistance [5].

Standard microbiological methods are constrained by slow turnaround times and reduced ability to detect atypical, fastidious, or polymicrobial infections in real-world practice [6]. Molecular approaches, including targeted assays and metagenomic next-generation sequencing (mNGS), can broaden pathogen detection in critically ill children and in LRTI more broadly [6, 7]. In parallel, host transcriptomic profiling provides complementary insight into immune activation patterns that can help differentiate the underlying aetiology of acute respiratory illness [8, 9]. Integrating host and microbial signals therefore offers a promising strategy to improve diagnostic accuracy in critical illness.

Recent work has demonstrated the potential of combined host–microbe profiling for LRTI classification in critically ill children, including mechanically ventilated PICU populations [10], and analogous approaches in critically ill adults [11]. However, important gaps remain in understanding how airway microbial profiles relate to host immune signalling networks in ventilated children with suspected LRTI, and in the exploratory evaluation of clinically deployable biomarker readouts in independent paediatric cohorts with detailed clinical adjudication [12]. Deeper exploration of host–microbe interactions and independent cohort assessment are essential steps towards actionable diagnostic biomarkers.

To address these gaps, we performed an integrated analysis of microbial mNGS data and host transcriptomic responses in the Mick et al. cohort of 261 critically ill children with acute respiratory failure [10]. Using differential expression analysis, protein–protein interaction networks, and machine-learning feature selection, we sought to characterise host–microbe relationships associated with paediatric LRTI and prioritise candidate early diagnostic biomarkers. We then performed an exploratory assessment of biologically related cytokine markers in an independent prospective cohort of ventilated children (RASCALS) [12], incorporating non-bronchoscopic bronchoalveolar lavage, blood cytokine profiling, and clinician-adjudicated diagnoses.

## Methodology

### Study design and participants

We obtained publicly available transcriptomic and clinical data from Mick et al. [10], which included a prospective cohort of 261 mechanically ventilated children (31 days–17 years) admitted to eight PICUs within the National Institute of Child Health and Human Development’s Collaborative Paediatric Critical Care Research Network (2015–2017). The dataset comprised tracheal aspirate RNA-sequencing and associated clinical metadata from patients meeting the inclusion criteria of requiring invasive mechanical ventilation via an endotracheal tube for ≥ 72 h. Further details on patient recruitment, ethical approvals, and sample collection procedures are available in Mick et al. [10].

For the independent exploratory cohort, we obtained data from the Rapid Assay for Sick Children with Acute Lung Infection Study (RASCALS [12]), a prospective cohort of 100 mechanically ventilated children under 18 years of age with suspected LRTI at Addenbrooke’s Hospital PICU in Cambridge, UK. Samples were obtained via non-bronchoscopic bronchoalveolar lavage (mini-BAL) and peripheral blood within 24 h of enrolment for pathogen detection and for cytokine profiling. Final LRTI status was assigned retrospectively based on the primary diagnosis at hospital discharge, with diagnoses of bronchiolitis, pneumonia, pneumonitis, or other lower respiratory tract infections classified as LRTI, and all other diagnoses considered non-LRTI. Further details on patient recruitment, ethical approvals, and sample collection procedures are available in *Clark et al.* [12].

### LRTI adjudication

LRTI diagnosis was adjudicated independently of mNGS results using (1) retrospective clinician review of all available clinical data and (2) standard-of-care respiratory microbiology from the first 48 h of intubation [8, 11]. Patients were categorised as Definite (clinical and microbiologic evidence), Suspected (clinical diagnosis without microbiologic findings), Indeterminate (microbiologic findings without a diagnosis), or No Evidence (alternative non-infectious cause). For this study, we defined *Definite* cases as LRTI and *No Evidence* cases as controls.

### Differential expression and microbial abundance analysis

Differential gene expression and differential microbial abundance were assessed using *DESeq2* [13] (version 1.46.0) and *edgeR* [14] (version 4.4.2), with normalisation via the median-of-ratios and TMM methods, respectively. These two packages are established, complementary methods for analysing count-based sequencing data, with differences in normalisation, dispersion estimation, and statistical testing. To minimise method-specific false positives and increase the robustness of our results, only features identified as significant by both methods were retained for downstream interpretation.

Low-expression genes were filtered by excluding genes with fewer than 10 counts in less than 20% of samples prior to differential expression analysis. Genes were defined as differentially expressed based on a Benjamini-Hochberg adjusted *P* < 0.05 and absolute log₂ fold change > 2. Only genes consistently identified as significant by both *DESeq2* and *edgeR* were retained as final differentially expressed genes. Microbial abundance was analysed using *DESeq2*’s “poscounts” and edgeR’s TMMwsp methods, with significance at *P* < 0.1.

### Protein network construction and analysis

Differentially expressed genes were mapped to proteins using Ensembl Biomart [15]. Protein-protein interaction networks were constructed using the STRING database [16] with a confidence threshold of > 0.4. Proteins not directly encoded by differentially expressed genes but connecting DEGs within the protein-protein interaction network (‘hidden layer proteins’) were included to capture intermediary proteins mediating host responses. Network centrality measures (eigenvector, degree, and betweenness centrality) and random walk with restart (RWR) were applied, with significance assessed via 1,000 permutations (*P* < 0.01).

### Feature selection and machine learning

Key proteins were mapped to gene IDs and analysed via LASSO logistic regression. The tuning parameter (λ) was selected by 5-fold cross-validation using the 1-standard-error criterion, which favours the most parsimonious model within 1 standard error of the minimum cross-validation error. Genes with non-zero coefficients in the final model were retained. For classifier development, host gene count data were transformed using the variance-stabilizing transformation implemented in *DESeq2* [13]. Binary LRTI classifiers (Logistic Regression, Random Forest, SVM, k-NN, XGBoost) were trained with 5-fold cross-validation. Hyperparameters were optimised for ROC performance.

### Pathway analysis

Signalling pathway impact analysis [17] (version 2.58.0) was conducted using the KEGG pathways provided within the package, incorporating over-representation and pathway perturbation metrics. Logistic regression classifiers were developed for each pathway, ranked by cross-validated F1 scores.

### Meta-analysis of RSV studies

GEO datasets [18] were screened up to August 2024 using the PICO framework [19]: the population (P) comprised children with respiratory infection and age-matched controls; the intervention (I) was RSV infection; the comparator (C) was healthy individuals within the same study population; and the measured outcome (O) was microarray-derived gene expression. Differential expression was analysed via GEO2R. Differentially expressed genes consistently identified in at least two of the four RSV studies were selected for integration into the meta-analysis.

### Cytokine classifier development (RASCALS exploratory cohort)

In the RASCALS exploratory cohort [12], we measured 48 cytokines in mini-BAL and plasma using the Bio-Plex Pro Human Cytokine Screening 48-plex kit. Clinical data were adjudicated by three PICU consultants to determine the presence of LRTI and classify infections as bacterial, viral, mixed or non-LRTI. For analysis, bacterial LRTI included both bacterial and mixed infections, non-bacterial LRTI referred to viral-only cases, and non-LRTI included patients without evidence of infection.

Logistic regression classifiers were trained separately using inverse-rank normalised mini-BAL and blood cytokine concentrations to distinguish bacterial LRTI from non-bacterial patients. Cut-offs were determined using the ‘closest to top-left’ criterion from receiver operating characteristic (ROC) curves. RSV status was incorporated into combined models for final performance evaluation.

### Statistical analysis

Differential expression and network significance were assessed as described above. Welch’s ANOVA was used to assess overall differences in performance among LRTI diagnostic models; where appropriate, Tukey’s post hoc test was used for pairwise comparisons within each algorithm type. Welch’s t-test was used to compare the abundance levels of identified microbial features between LRTI and control samples. Kendall’s correlation was applied to normalised counts of RSV and bacterial or fungal species to identify positive co-occurrence patterns. A significance threshold of *P* < 0.05 was used for hypothesis tests, and *P* < 0.1 was used for correlation analyses.

### Data and code availability

All data, code, and software packages used, including versions and citations, are available at https://github.com/zongtaiwu/mphil-paediatric-LRTI. The raw data, including patient adjudication, host gene counts, and microbial taxon counts were retrieved from *Mick et al.* [10] at https://github.com/eranmick/pediatric-mNGS-LRTI-classifier. The code for protein network analysis was adapted based on the code from Han et al. [20] at https://github.com/wchwang/COVID19.

## Results

### Dataset and patient selection

We first analysed microbial and host gene expression data [10] from 261 children with acute respiratory failure admitted to PICU. Patients were categorised as Definite LRTI (*n* = 117), No Evidence of LRTI (*n* = 50), Suspected (*n* = 57), or Intermediate (*n* = 37). For primary analyses, Definite cases and No Evidence controls were included, while Suspected and Intermediate groups were excluded because of diagnostic uncertainty.

For further exploratory assessment, we examined a prospective cohort of 100 critically ill children with suspected LRTI (RASCALS [12]). Each patient underwent non-bronchoscopic bronchoalveolar lavage (mini-BAL) and blood sampling for pathogen detection and cytokine profiling. Final diagnosis of LRTI or non-LRTI was assigned at hospital discharge and adjudicated by consultant consensus including classification as bacterial or non-bacterial infection. Clinical outcomes were recorded, with ventilator-free days at 28 days post-admission used as a marker of severity. Figure SF1 summarises patient selection and study design.

### Key host genes, proteins, and pathways associated with LRTI progression

**Fig. 1:**
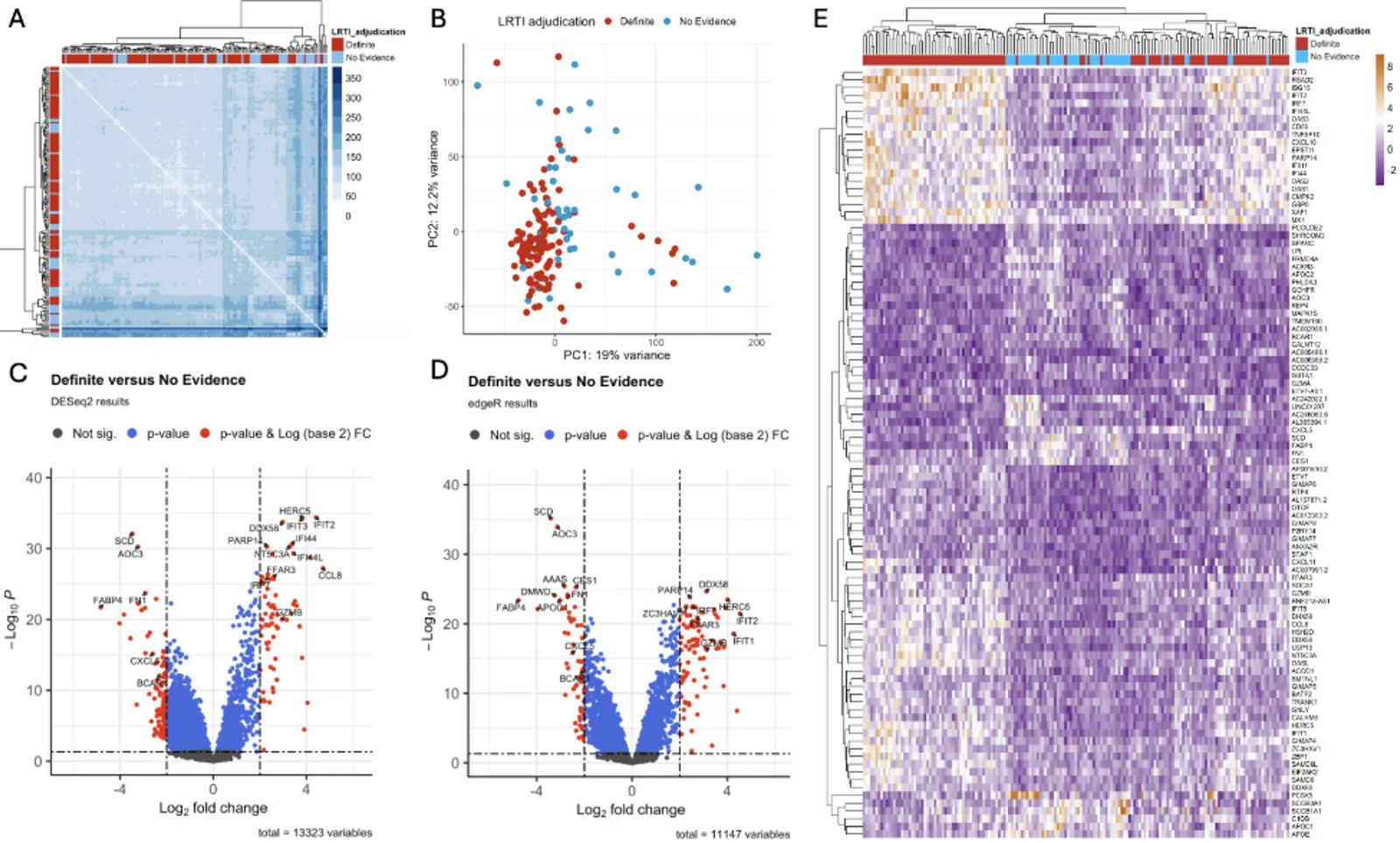
**A** Heatmap of sample-to-sample Euclidean distances in LRTI samples and controls using the variance stabilising transformed (VST) values. **B** PCA plot in in LRTI samples and controls using the VST values. **C** Volcano plot of differential expression analysis between LRTI and control cohorts using DESeq2. FDR adjusted *p*-value < 0.05, and absolute log2 fold change > 2 was deemed significant. **D** Volcano plot using edgeR. **E** Heatmap of VST values across all patients from Definite and No Evidence (columns) for the 99 differential expressed genes intersecting from DESeq2 and edgeR (rows). LRTI adjudication (top coloured horizontal bar) is displayed alongside the regression coefficients for each selected gene (side bar plot)

After filtering low-expression genes, 13,323 transcripts were retained for host transcriptomic analysis. Unsupervised clustering and principal component analysis (PCA) of variance-stabilized expression values showed broad differences in global expression structure between LRTI cases and controls, although separation was not complete (Figs. 1A–B). Differential expression analysis identified 180 genes by DESeq2 and 144 by edgeR, with 99 consistently detected by both methods (Figs. 1C–D). These shared genes exhibited distinct expression patterns between groups, and hierarchical clustering showed clear case–control grouping (Fig. 1E).

**Fig. 2:**
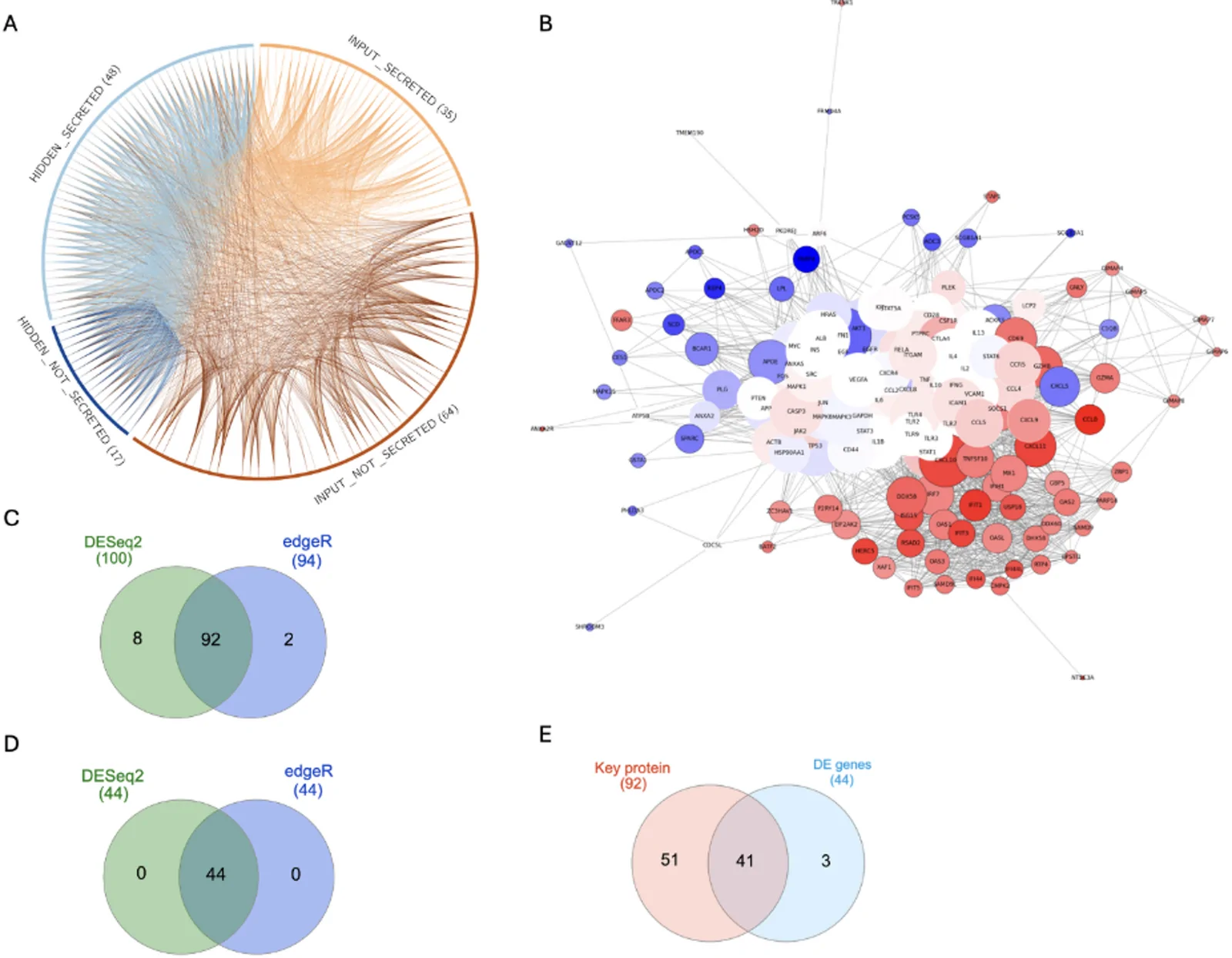
**A** A circus plot depicting interactions between DEPs through the PPI network. The plot includes only input and key proteins, where 65 key proteins are in the hidden layer. The proteins are subdivided based on their secretion into bloodstream. The coloured lines show PPI. **B** A plot showing the network which includes only input and key proteins. Nodes with larger sizes have higher eigenvector centrality. The node colour reflects the log2 fold change in gene expression, with red indicating positive values, blue indicating negative values, and white indicating near-zero or N/A values. **C** Number of pathways from SPIA using DESeq2 and edgeR results on key protein genes. **D** Number of pathways from SPIA using DESeq2 and edgeR results on DEGs. **E** Intersection of pathways in (**C**) and (**D**)

To assess functional relationships, we constructed a protein–protein interaction (PPI) network using differentially expressed proteins (DEPs) encoded by the 99 differentially expressed genes (DEGs) (Fig. 2A). Using network centrality metrics, we identified 88 centrally positioned key network proteins (Fig. 2B), suggesting key roles in host immune responses during LRTI. The corresponding network centrality results are provided in Supplementary Table 2. Pathway enrichment analysis revealed 41 consistently perturbed immune cascades, including cytokine, chemokine, Toll-like receptor, and interferon signalling (Figs. 2C–E). These enrichments were driven by proteins involved in host inflammatory and antiviral responses, with the pathway-member annotations provided in the Supplementary Tables 4–8. Overall, LRTI was characterised by enrichment of immune and host defence pathways and activation of central network proteins likely driving disease progression.

### LRTI diagnostic markers and disease pathways selected by supervised learning methods

**Fig. 3:**
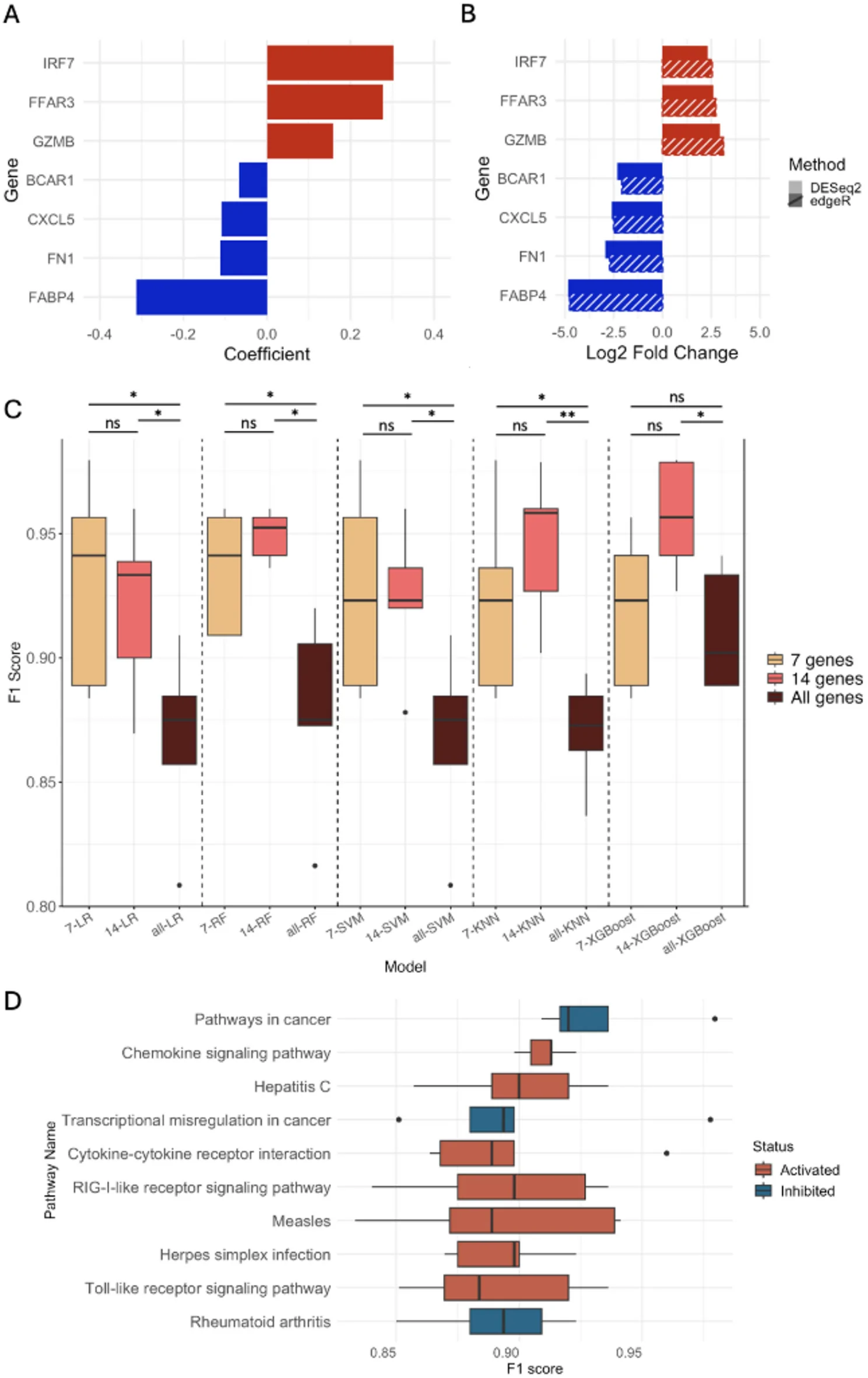
**A** Regression coefficients of the 7 diagnostic markers selected from LASSO logistic regression model. **B** Log_2_ fold change of the 7 diagnostic markers from differential expression analysis. **C** Different models for classification of LRTI status. Boxplot shows F1 scores across 5-fold cross- validation. Significance levels of two-tailed Tuckey’s tests are indicated as follows: *P < 0.05, **P < 0.01, and “ns” for not significant. LR: logistic regression; RF: random forest; SVM: support vector machine; KNN: k-nearest neighbors; XGBoost: eXtreme Gradient Boosting. **D** Top 10 LR classifiers using key genes in each pathway. Boxplot shows F1 scores across 5-fold cross-validation. Pathway names correspond to standard KEGG annotations. Disease-labelled pathways (e.g., rheumatoid arthritis) are interpreted here as reflecting shared underlying inflammatory and immune signalling components rather than disease-specific mechanisms

From 88 key network proteins, we derived a seven-gene diagnostic panel that distinguished LRTI cases from controls using LASSO logistic regression. The genes IRF7, FFAR3, and GZMB were upregulated, while FABP4, FN1, CXCL5, and BCAR1 were downregulated (Figs. 3A–B). Two genes (CXCL5 and FABP4) overlapped with the previously reported 14-gene classifier [10], while five were unique to this study.

The seven-gene panel showed high diagnostic performance, with a median F1 score of 0.94 across logistic regression, random forest, and other classifiers, comparable to the 14-gene model (Fig. 3C). Performance did not differ significantly between the two panels, and both clearly outperformed models trained on the full unfiltered gene set. Thus, the streamlined seven-gene panel offers accuracy comparable to the 14-gene model but with greater practicality.

Pathway-level classifier analysis showed that genes grouped within immune-related pathways, particularly chemokine, cytokine–receptor, and Toll-like receptor signalling, achieved the strongest diagnostic performance for distinguishing LRTI cases from controls (Fig. 3D; Table 1). These results indicate that both discrete host gene signatures and broader immune pathways provide candidate biomarkers for early LRTI diagnosis in critically ill children.

**Table 1:** Key protein genes in the top 10 pathways. The table shows the gene symbols along with their log2 fold change values from DESeq2

Gene	Log_2_ Fold Change	Gene	Log_2_ Fold Change
*CXCL11*	3.8087	*FN1*	-2.9120
*CXCL10*	3.7020	*CXCL5*	-2.5964
*RIGI*	2.9773	*BCAR1*	-2.2964
*GZMB*	2.9406	*CXCL8*	-1.1641
*TNFSF10*	2.5170	*AKT1*	-0.6730
*IRF7*	2.3306	*TP53*	-0.6053
*EIF2AK2*	2.1516	*JUN*	-0.4068
*CXCL9*	2.0545	*MAPK8*	-0.3361
*CTLA4*	1.5173		
*STAT1*	1.1565		
*CCL5*	0.9493		
*JAK2*	0.8516		
*CCR5*	0.8254		
*CCL4*	0.7529		
*RELA*	0.6073		
*CASP3*	0.5549		
*ICAM1*	0.5263		
*IL10*	0.4322		
*ITGAM*	0.4129		
*TLR4*	0.3881		
*TLR2*	0.3472		

### Key microbial strains associated with LRTI progression

**Fig. 4:**
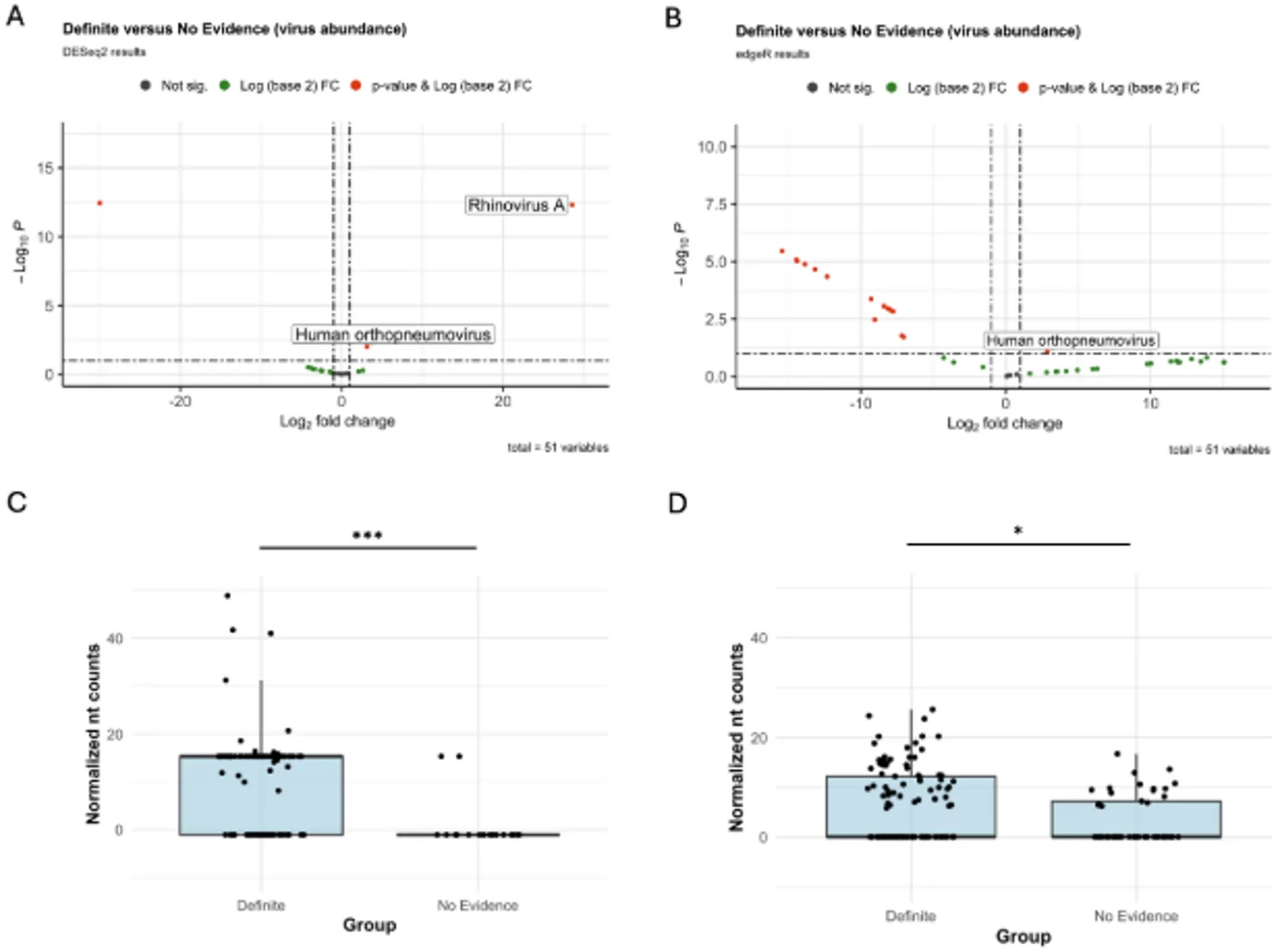
**A** Volcano plot of differential analysis between LRTI and control cohorts on virus abundance using DESeq2. P-value < 0.1 and log_2_ fold change > 1 was deemed significant. **B** Volcano plot on virus abundance using edgeR. Scatter bar chart representing abundance of (**C**) Respiratory Syncytial Virus and (**D**) Haemophilus influenzae between LRTI and control patients. Significance levels of two-tailed Welch’s two-sample t-test are indicated as follows: *P < 0.05, ***P < 0.001

We next examined dominant microbial features associated with LRTI. To control for contaminants, microbial counts were adjusted against water controls. After filtering, viruses were detected in 91% of LRTI samples (107/117) versus 16% of controls (8/50), spanning 51 viral species. Differential abundance analysis identified respiratory syncytial virus (RSV) by both DESeq2 and edgeR, while rhinovirus A was detected only by DESeq2 (Figs. 4A–B). RSV was therefore the sole virus consistently enriched in LRTI.

For bacteria and fungi, classification at the genus level showed Haemophilus and Moraxella enriched by both methods (Figures SF5-6). At the species level, only *Haemophilus influenzae* was consistently identified across DESeq2 and edgeR (Figures SF7-8). Subsequent analyses therefore focused on *H. influenzae*.

Quantitative comparisons using the Mann-Whitney U test confirmed RSV and *H. influenzae* enrichment in LRTI: RSV (*P* < 0.001, Fig. 4C) and *H. influenzae* (*P* = 0.014, Fig. 4D). Among LRTI samples, RSV was present in 67% (72/107) and *H. influenzae* in 49% (57/116). Given RSV’s dominance, we explored its co-occurrence patterns with other microbial species using Kendall’s rank test. RSV co-occurred with five additional taxa (Supplementary Table 9), including *T. atroviride*, *H. influenzae*, and several other bacterial species. These findings identify RSV and *H. influenzae* as microbial features associated with LRTI, and the strong correlation between RSV and *H. influenzae* highlights a potential synergistic interaction exacerbating LRTI severity.

### RSV-associated host gene expression and network perturbations underpin LRTI pathogenesis

**Fig. 5:**
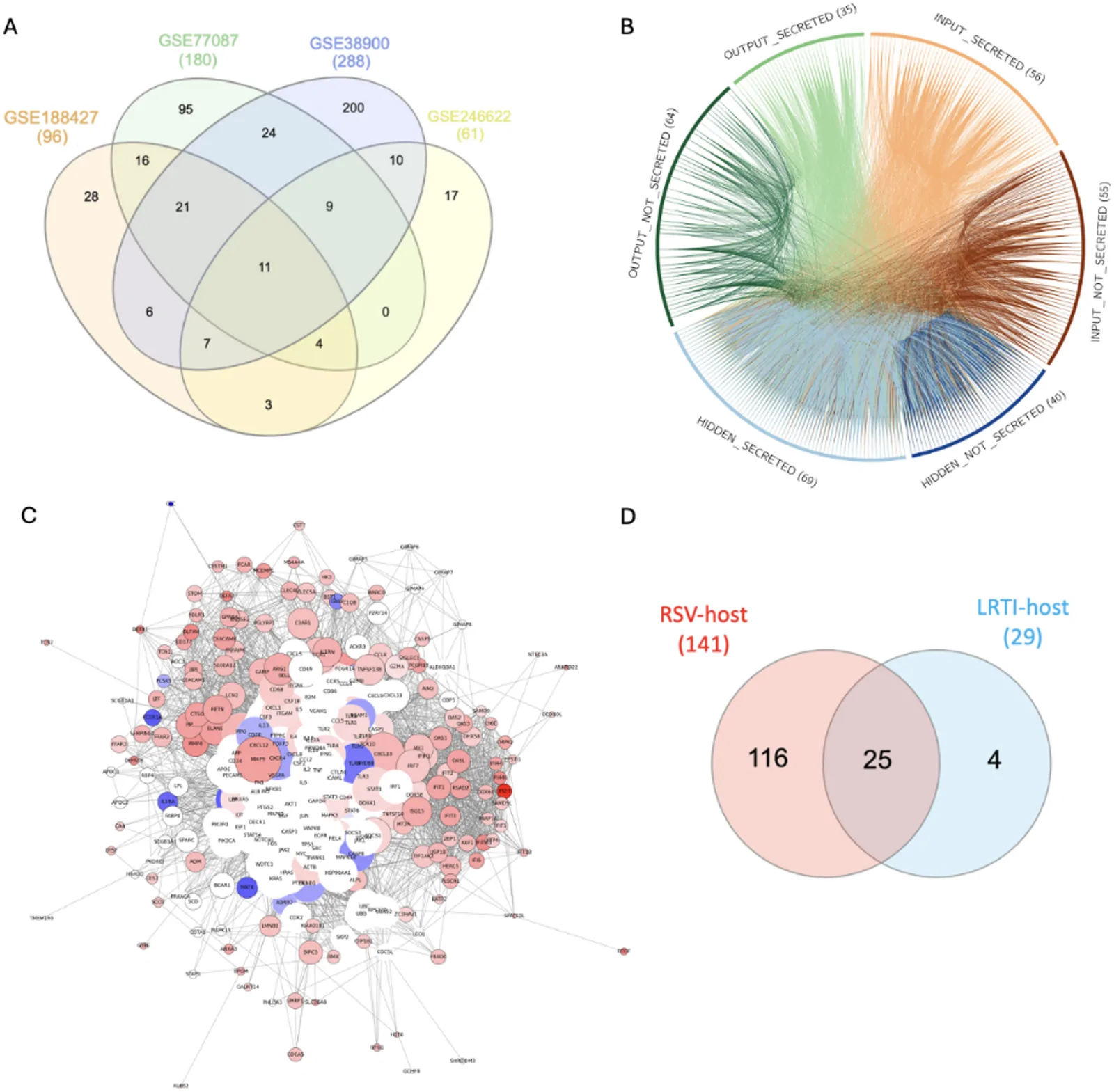
**A** A Venn diagram showing the number of overlapping differentially expressed genes associated with RSV across four studies. **B** A circus plot depicting interactions between input and output proteins through the PPI network. The plot includes input, output and key proteins, where 109 key proteins are in the hidden layer. The proteins are subdivided based on their secretion into bloodstream. The coloured lines show PPI. **C** A plot showing the network which includes only input and key proteins. Nodes with larger sizes have higher eigenvector centrality. The node colour reflects the log_2_ fold change in gene expression, with red indicating positive values, blue indicating negative values, and white indicating near-zero or N/A values. **D** A Venn diagram showing key protein genes identified in the top 10 pathways and central in the RSV-host network

Given RSV’s dominance, we assessed its impact on host responses through a meta-analysis of four independent transcriptomic studies of RSV-infected children (Supplementary Table 10). Using differential expression analysis, 111 genes were consistently identified as differentially expressed across at least two studies (Fig. 5A), indicating conserved host responses to RSV.

Protein-protein interaction (PPI) network analysis showed that RSV-associated genes clustered in immune and inflammatory signalling pathways relevant to LRTI pathogenesis. Integrating RSV-specific DEGs with LRTI-associated DEGs produced an RSV–host interaction network containing 141 central proteins (Figs. 5B–C). Many of these acted as intermediary “hidden layer” proteins linking RSV-induced responses to broader immune dysregulation.

Overlap analysis demonstrated that 25 of 29 key host proteins involved in the most significantly perturbed immune pathways in LRTI (Table 1) were also central in the RSV–host network (Fig. 5D; Supplementary Table 11). This high degree of overlap highlights RSV as a primary driver of inflammatory signalling in paediatric LRTI.

Overall, these results suggest that RSV infection triggers conserved host gene networks that amplify immune activation and underpin LRTI progression.

### Cytokine-linked key network proteins underpin bacterial LRTI pathogenesis

We evaluated candidate biomarkers in an independent prospective cohort of 100 critically ill children with suspected LRTI (RASCALS [12]). Of these, 78 patients were included in the analysis after excluding those without available samples or with insufficient remaining material following priority experiments. The median age was 1.2 years (IQR 0.4–5.2 years). Mini-bronchoalveolar lavage (mini-BAL) and plasma blood samples were analysed alongside clinical outcomes, with ventilator-free days at 28 days serving as the primary severity marker.

**Fig. 6:**
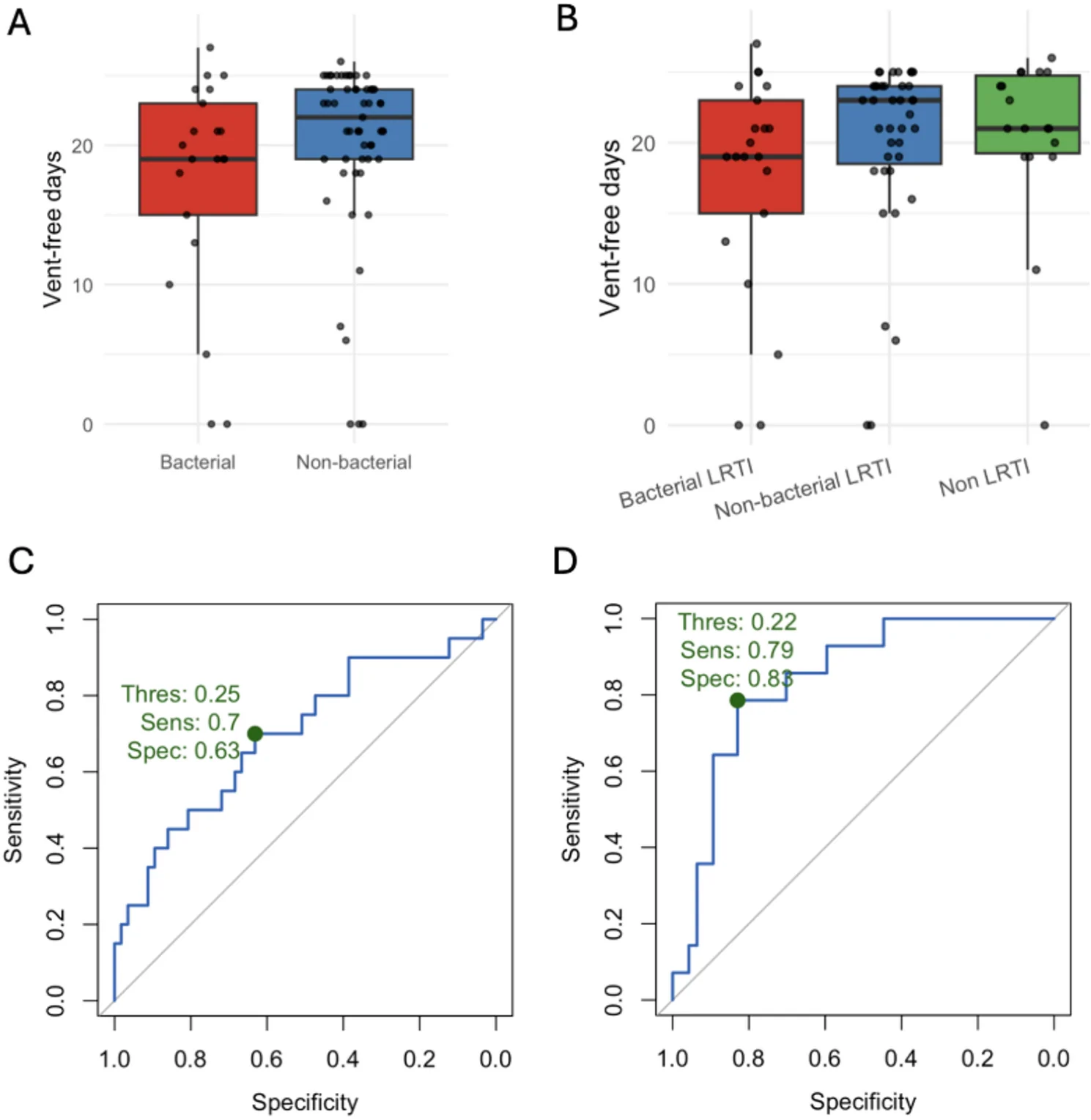
Distribution of ventilator-free days at 28 days following PICU admission (**A**) between bacterial LRTI and non-bacterial groups; (**B**) among bacterial LRTI, non-bacterial LRTI, and non LRTI groups. Receiver-operating characteristic (ROC) curves for logistic-regression model predicting LRTI status. **C** Model using mini-BAL IL-1β + IL-4 + IL-8. **D** Model using blood IL-6 + TRAIL. In each panel, the green dot marks the cut-off probability that yields the point on the curve closest to the top-left corner (optimal trade-off), with the corresponding threshold (Thres), sensitivity (Sens), and specificity (Spec) annotated. AUC, area under the ROC curve

Clinical data were reviewed by three PICU consultants to adjudicate LRTI status and classify infections as bacterial, non-bacterial, or non-LRTI. Children with bacterial LRTI (*n* = 21) had fewer ventilator-free days compared to the non-bacterial group (*n* = 57), suggesting greater disease severity and higher intervention needs, though the difference did not reach statistical significance (*P* = 0.10; Fig. 6A). When the non-bacterial group was further subdivided into viral LRTI (*n* = 39) and non-LRTI (*n* = 18), bacterial LRTI again showed the poorest outcomes, although without statistical significance (*P* = 0.24, Fig. 6B). These findings highlight the potential value of improved discrimination between bacterial and non-bacterial LRTI, which may help support more appropriate and targeted antimicrobial decision-making in critically ill children.

Respiratory syncytial virus (RSV) was detected in 11 patients, all of whom were diagnosed with LRTI, consistent with previous findings identifying RSV as a key risk factor. Of these, 10 were classified as viral LRTI and one as mixed infection.

As a secondary exploratory analysis in the independent RASCALS cohort, we examined host responses associated specifically with bacterial LRTI. Five cytokine markers were selected from the key host proteins identified in our network analysis on the basis that they were measurable in the available RASCALS mini-BAL or blood cytokine datasets: mini-BAL IL-1β (IL1B), IL-4, and IL-8 (CXCL8), and blood IL-6 and TRAIL (TNFSF10). Mini-BAL IL-1β, IL-4, and IL-8 levels were significantly elevated in bacterial LRTI compared to non-bacterial cases (*P* = 0.022, 0.027, and 0.019, respectively; Figures SF12–13). Similarly, plasma IL-6 levels were significantly higher in bacterial LRTI (*P* = 0.001), while TRAIL levels were significantly lower (*P* = 0.049; Figures SF12–13). Logistic regression models combining these cytokines achieved moderate-to-high diagnostic performance, with mini-BAL IL-1β + IL-4 + IL-8 achieving 65% diagnostic accuracy (sensitivity 70%, specificity 63%; Fig. 6C, Table 2), and blood IL-6 + TRAIL achieving 82% accuracy (sensitivity 79%, specificity 83%; Fig. 6D, Table 3).

**Table 2:** Mini-BAL IL-1β + IL-4 + IL-8 prediction versus clinical diagnosis of bacterial LRTI in the RASCALS cohort. 77 out of 78 patients have mini-BAL cytokine data

	Diagnosed Bacterial LRTI	Diagnosed Non-bacterial
Predicted Bacterial LRTI	14	21
Predicted Non-bacterial	6	36

**Table 3:** Blood IL-6 + TRAIL prediction versus clinical diagnosis of LRTI in the RASCALS cohort. 61 out of 78 patients have blood cytokine data

	Diagnosed Bacterial LRTI	Diagnosed Non-bacterial
Predicted Bacterial LRTI	11	8
Predicted Non-bacterial	3	39

Together, these results demonstrate that bacterial LRTI is associated with greater clinical severity, and that specific cytokine profiles can aid in distinguishing bacterial infections from non-bacterial causes in critically ill children.

## Discussion

This study shows that combining host-response biomarkers with airway microbial features can support early diagnostic classification of paediatric LRTI in critically ill children. Using metagenomic airway sequencing from a multicentre PICU cohort, we identified a streamlined seven-gene host signature and microbial features that were strongly associated with adjudicated LRTI. In an independent prospective cohort (RASCALS [12]), we then evaluated clinically deployable inflammatory readouts and observed that blood cytokines achieved 82% accuracy for bacterial LRTI, while mini-BAL cytokines achieved 65% accuracy. Together, these findings support a translational framework in which host–microbe profiling can contribute to the development of practical biomarker strategies with promising diagnostic accuracy in PICU settings.

Compared with the 14-gene host-response signature reported by Mick et al. [10], our seven-gene panel achieved comparable performance in the reanalysed dataset while offering greater simplicity and clearer biological interpretability. Reducing panel complexity is important for translation into rapid PCR-based assays in acute care settings. Standard diagnostics, including culture and multiplex PCR, can detect organisms but do not reliably distinguish infection from colonisation, and they provide limited information on the host inflammatory state that often determines clinical trajectory. Commonly used host biomarkers such as C-reactive protein and procalcitonin can be informative but are not sufficiently specific to consistently distinguish bacterial from viral aetiologies in critically ill children. An integrated approach that couples pathogen detection with host immune profiling may therefore better support clinically relevant classification of LRTI.

The seven-gene host panel (IRF7, FFAR3, GZMB, FABP4, FN1, CXCL5, BCAR1) demonstrated strong diagnostic performance and biological plausibility. IRF7, GZMB, and CXCL5 are central to antiviral and inflammatory signalling [21–23], whereas FFAR3 and FABP4 relate to immunometabolic regulation [24, 25], and FN1 and BCAR1 have roles in epithelial structure and repair [26, 27]. The prominence of immune activation genes is consistent with the idea that host-response patterns can provide a stable and discriminatory signal, particularly in settings where pathogen detection may reflect a mixture of infection, colonisation, and background airway microbiota. This aligns with broader evidence that severe LRTI is strongly influenced by host immune dysregulation, not pathogen burden alone.

Microbial analyses highlighted RSV and *H. influenzae* as key microbial features, consistent with established epidemiological patterns in paediatric respiratory disease [28, 29]; however, the present analysis did not distinguish typable from non-typable *H. influenzae*. The co-occurrence of viral and bacterial signatures is also consistent with longstanding clinical observations that viral infection can predispose to secondary bacterial complications. Mechanistic work suggests that viral infection can impair mucosal defence and alter epithelial integrity, increasing susceptibility to bacterial overgrowth and inflammation [30]. In our network analysis, we prioritised host genes that connect viral-associated immune responses with bacterial-associated inflammatory pathways, providing a molecular context for viral–bacterial interaction in critically ill paediatric patients. While causality cannot be inferred from these observational data, the convergence of host and microbial signals supports the biological plausibility of this cascade.

In RASCALS, blood cytokines outperformed mini-BAL cytokines. This may indicate that systemic immune signatures better reflect clinically adjudicated infection status than local airway inflammation, which can be influenced by mechanical ventilation, sampling variability, and compartmentalised responses. From an implementation perspective, blood-based biomarkers may also be more scalable for routine PICU use, whereas mini-BAL sampling is not universally performed and may not be feasible in all centres.

Pathway analyses reinforced the centrality of immune activation in LRTI biology, including chemokine signalling, cytokine–receptor interactions, RIG-I-like receptor signalling, and Toll-like receptor pathways. The relative downregulation of pathways related to proliferation and longer-term cellular programmes is consistent with acute reprioritisation towards innate antiviral defence during critical illness. These findings support a model in which immune activation state is a key determinant of clinical phenotype, and thus a rational target for biomarker development.

These results have direct relevance for antimicrobial stewardship in PICU. Diagnostic uncertainty often drives empirical broad-spectrum antibiotic use, increasing antimicrobial resistance risk and exposing vulnerable children to unnecessary treatment. A blood-based classifier (for example, IL-6 and TRAIL), if shown in future studies to provide reliable discriminatory information within an actionable timeframe, could support earlier de-escalation in viral-predominant presentations and more timely escalation when bacterial infection is strongly suspected. Importantly, future evaluations should move beyond accuracy metrics to quantify whether biomarker-guided decisions reduce antibiotic exposure without compromising safety.

Translation of these biomarkers is feasible but requires additional steps. The seven-gene panel could be adapted to rapid qPCR workflows, and cytokine assays could be implemented using clinically validated immunoassay platforms. However, before clinical deployment, prospective multicentre validation with locked models, pre-specified decision thresholds, and assessment of calibration and clinical utility will be essential, alongside health-economic evaluation.

This study has limitations. The discovery analyses rely on a single public cohort and primary analyses focus on “Definite” versus “No Evidence” labels, which may overestimate performance relative to the diagnostically uncertain cases that dominate real-world decision-making. In addition, the seven-gene host panel was developed for classification of LRTI versus non-LRTI and does not by itself resolve the clinically important distinction between bacterial and viral LRTI that underpins antimicrobial stewardship in PICU. The RASCALS cohort serves as an exploratory assessment of biologically related cytokine markers rather than a like-for-like external validation of the seven-gene transcriptomic assay. Functional experiments would be required to test the mechanistic hypotheses suggested by network analyses. Microbial resolution and taxonomic assignment may also obscure strain-level effects, and imbalance in pathogen distributions limited incorporation of microbial load into unified classifier models. Finally, multicentre validation across diverse populations and care pathways is needed to assess generalisability and real-world impact on antibiotic use and outcomes.

In summary, this study identifies a simplified host-response gene signature and complementary microbial features associated with paediatric LRTI in mechanically ventilated children, and provides prospective evidence that blood cytokine signatures can support bacterial–nonbacterial discrimination in PICU. By linking microbial detection with host immune profiling, we outline a translational route towards more precise, actionable diagnostics to support antimicrobial stewardship in paediatric respiratory failure.

## Conclusion

This study shows that integrating host-response and microbial features can support earlier diagnostic classification of paediatric LRTI in critically ill, mechanically ventilated children. In a multicentre PICU discovery cohort, we identified a simplified seven-gene host signature alongside key microbial signals (including RSV and *Haemophilus influenzae*) associated with adjudicated LRTI, and in an exploratory assessment of an independent cohort we observed that blood- and airway-derived cytokine signatures (including IL-6 and TRAIL) provided clinically relevant discrimination of bacterial LRTI.

These findings support further development of rapid PCR- and immunoassay-based biomarker tests to inform antimicrobial decision-making in PICU. Prospective multicentre validation with locked models and pre-specified thresholds, together with assessment of clinical utility and implementation, is now required before routine clinical use.

## Supplementary Information

Supplementary Material 1.

Supplementary Material 2.

## Data Availability

All data, code, and software packages used, including versions and citations, are available at https://github.com/zongtaiwu/mphil-paediatric-LRTI. The raw data, including patient adjudication, host gene counts, and microbial taxon counts were retrieved from *Mick et al. [10] at https:/github.com/eranmick/pediatric-mNGS-LRTI-classifier. The code for protein network analysis was adapted based on the code from Han et al.[20] at https://github.com/wchwang/COVID19. This study includes secondary analysis of clinical samples and data collected under the Rapid Assay for Sick Children with Acute Lung Infection Study (RASCALS). The original RASCALS study received ethical approval from the Yorkshire and the Humber, Bradford Leeds Research Ethics Committee (Research Ethics Committee reference 20/YH/0089), under the governance of the UK Health Research Authority (HRA). Ethical approval was granted for prospective participant recruitment, sample collection, and the use of routine and research-based diagnostic assays. Deferred consent for up to 48 h was permitted in accordance with the approved protocol. For the current analysis, all samples and associated clinical data were anonymised prior to inclusion, and therefore no additional ethical approval was required. Informed consent was obtained from parents or legal guardians of all participating children enrolled in the RASCALS study.I confirm that all necessary patient/participant consent has been obtained and the appropriate institutional forms have been archived, and that any patient/participant/sample identifiers included were not known to anyone (e.g., hospital staff, patients or participants themselves) outside the research group so cannot be used to identify individuals. Clinical trial number: not applicable.

## Abbreviations

DEG: Differentially expressed gene
DEP: Differentially expressed protein
k-NN: K-nearest neighbors
LRTI: Lower respiratory tract infection
mini-BAL: Non-bronchoscopic bronchoalveolar lavage
mNGS: Metagenomic next-generation sequencing
ROC: Receiver operating characteristic
PCR: Polymerase chain reaction
PICU: Paediatric intensive care unit
RSV: Respiratory syncytial virus
SVM: Support vector machine
XGBoost: EXtreme Gradient Boosting

## References

[1] GBD 2016 Causes of Death Collaborators. Global, regional, and national age-sex specific mortality for 264 causes of death, 1980–2016: a systematic analysis for the Global Burden of Disease Study 2016. Lancet 2017;390(10100):1151–210. 10.1016/S0140-6736(17)32152-9 PMC5605883 28919116

[2] GBD 2023 Lower Respiratory Infections and Antimicrobial Resistance Collaborators. Global burden of lower respiratory infections and aetiologies, 1990–2023: a systematic analysis for the Global Burden of Disease Study 2023. Lancet Infect Dis. 2026;26(4):343–61. 10.1016/S1473-3099(25)00689-9. 41412141

[3] Jain S, Williams DJ, Arnold SR, Ampofo K, Bramley AM, Reed C, et al. Community-acquired pneumonia requiring hospitalization among U.S. children. N Engl J Med. 2015;372(9):835–45. 10.1056/NEJMoa1405870 PMC4697461 25714161

[4] Netea MG, Schlitzer A, Placek K, Joosten LAB, Schultze JL. Innate and adaptive immune memory: an evolutionary continuum in the host’s response to pathogens. Cell Host Microbe. 2019;25(1):13–26. 10.1016/j.chom.2018.12.006 30629914

[5] Goossens H, Ferech M, Stichele RV, Elseviers M. Outpatient antibiotic use in Europe and association with resistance: a cross-national database study. Lancet. 2005;365(9459):579–87. 10.1016/S0140-6736(05)17907-0 15708101

[6] Lv T, Zhao Q, Liu J, Wang S, Wu W, Miao L, et al. Utilizing metagenomic next-generation sequencing for pathogen detection and diagnosis in lower respiratory tract infections in real-world clinical practice. Infection. 2024;52(2):625–36. 10.1007/s15010-024-02185-1 38368306

[7] Clark JA, Conway Morris A, Curran MD, White D, Daubney E, Kean IRL, et al. The rapid detection of respiratory pathogens in critically ill children. Crit Care. 2023;27(1):11. 10.1186/s13054-023-04303-1 PMC9831374 36627688

[8] Tsalik EL, Henao R, Nichols M, Burke T, Ko ER, McClain MT, et al. Host gene expression classifiers diagnose acute respiratory illness etiology. Sci Transl Med. 2016;8(322):322ra11. 10.1126/scitranslmed.aad6873 PMC4905578 26791949

[9] Chaussabel D, Pascual V, Banchereau J. Assessing the human immune system through blood transcriptomics. BMC Biol. 2010;1(1):84. 10.1186/1741-7007-8-84 PMC2895587 20619006

[10] Mick E, Tsitsiklis A, Kamm J, Kalantar KL, Caldera S, Lyden A et al. Integrated host/microbe metagenomics enables accurate lower respiratory tract infection diagnosis in critically ill children. J Clin Invest. 2023;133(7). Available from: https://www.jci.org/articles/view/165904. Cited 31 Jul 2024. 10.1172/JCI165904 PMC10065066 37009900

[11] Langelier C, Kalantar KL, Moazed F, Wilson MR, Crawford ED, Deiss T et al. Integrating host response and unbiased microbe detection for lower respiratory tract infection diagnosis in critically ill adults. Proceedings of the National Academy of Sciences. 2018;115(52):E12353–62. 10.1073/pnas.1809700115 PMC6310811 30482864

[12] Clark JA, Kean IRL, Curran MD, Khokhar F, White D, Daubney E, et al. Rapid Assay for Sick Children with Acute Lung infection Study (RASCALS): diagnostic cohort study protocol. BMJ Open. 2021;11(11):e056197. 10.1136/bmjopen-2021-056197 PMC8634010 34845080

[13] Love MI, Huber W, Anders S. Moderated estimation of fold change and dispersion for RNA-seq data with DESeq2. Genome Biol. 2014;15(12):550. 10.1186/s13059-014-0550-8 PMC4302049 25516281

[14] Robinson MD, McCarthy DJ, Smyth GK. edgeR: a Bioconductor package for differential expression analysis of digital gene expression data. Bioinformatics. 2010;26(1):139–40. 10.1093/bioinformatics/btp616 PMC2796818 19910308

[15] Harrison PW, Amode MR, Austine-Orimoloye O, Azov AG, Barba M, Barnes I, et al. Ensembl 2024. Nucleic Acids Res. 2024;52(D1):D891–9. 10.1093/nar/gkad1049 PMC10767893 37953337

[16] Szklarczyk D, Gable AL, Lyon D, Junge A, Wyder S, Huerta-Cepas J, et al. STRING v11: protein-protein association networks with increased coverage, supporting functional discovery in genome-wide experimental datasets. Nucleic Acids Res. 2019;47(D1):D607–13. 10.1093/nar/gky1131 PMC6323986 30476243

[17] Tarca AL, Draghici S, Khatri P, Hassan SS, Mittal P, Kim JS, et al. A novel signaling pathway impact analysis. Bioinformatics. 2009;25(1):75–82. 10.1093/bioinformatics/btn577 PMC2732297 18990722

[18] Barrett T, Wilhite SE, Ledoux P, Evangelista C, Kim IF, Tomashevsky M, et al. NCBI GEO: archive for functional genomics data sets—update. Nucleic Acids Res. 2013;41(D1):D991–5. 10.1093/nar/gks1193 PMC3531084 23193258

[19] Schardt C, Adams MB, Owens T, Keitz S, Fontelo P. Utilization of the PICO framework to improve searching PubMed for clinical questions. BMC Med Inf Decis Mak. 2007;15:7:16. 10.1186/1472-6947-7-16 PMC1904193 17573961

[20] Han N, Hwang W, Tzelepis K, Schmerer P, Yankova E, MacMahon M, et al. Identification of SARS-CoV-2-induced pathways reveals drug repurposing strategies. Sci Adv. 2021;7(27):eabh3032. 10.1126/sciadv.abh3032 PMC8245040 34193418

[21] Honda K, Yanai H, Negishi H, Asagiri M, Sato M, Mizutani T, et al. IRF-7 is the master regulator of type-I interferon-dependent immune responses. Nature. 2005;434(7034):772–7. 10.1038/nature03464 15800576

[22] Voskoboinik I, Whisstock JC, Trapani JA. Perforin and granzymes: function, dysfunction and human pathology. Nat Rev Immunol. 2015;15(6):388–400. 10.1038/nri3839 25998963

[23] Guo L, Li N, Yang Z, Li H, Zheng H, Yang J, et al. Role of CXCL5 in regulating chemotaxis of innate and adaptive leukocytes in infected lungs upon pulmonary influenza infection. Front Immunol. 2021;12:785457. 10.3389/fimmu.2021.785457 PMC8637413 34868067

[24] Liu Q, Tian X, Maruyama D, Arjomandi M, Prakash A. Lung immune tone via gut-lung axis: gut-derived LPS and short-chain fatty acids’ immunometabolic regulation of lung IL-1β, FFAR2, and FFAR3 expression. American Journal of Physiology-Lung Cellular and Molecular Physiology. 2021;321(1):L65–78. 10.1152/ajplung.00421.2020 PMC8321849 33851870

[25] Perea L, Rodrigo-Troyano A, Cantó E, Domínguez-Álvarez M, Giner J, Sanchez-Reus F, et al. Reduced airway levels of fatty-acid binding protein 4 in COPD: relationship with airway infection and disease severity. Respir Res. 2020;21:21. 10.1186/s12931-020-1278-5 PMC6958639 31931795

[26] van Nijnatten J, Faiz A, Timens W, Guryev V, Slebos DJ, Klooster K et al. A bronchial gene signature specific for severe COPD that is retained in the nose. ERJ Open Research. 2023;9(6). Available from: https://openres.ersjournals.com/content/9/6/00354-2023. Cited 17 Jul 2024. 10.1183/23120541.00354-2023 PMC10680034 38020574

[27] del Maria CL, Marianna S, Giusy T, Shana C, Paola D, Sara C. p130Cas/BCAR1 scaffold protein in tissue homeostasis and pathogenesis. Gene. 2015;562(1):1–7. 10.1016/j.gene.2015.02.027 PMC6639054 25727852

[28] Shi T, McAllister DA, O’Brien KL, Simoes EAF, Madhi SA, Gessner BD et al. Global, regional, and national disease burden estimates of acute lower respiratory infections due to respiratory syncytial virus in young children in 2015: a systematic review and modelling study. The Lancet. 2017;390(10098):946–58. 10.1016/S0140-6736(17)30938-8 PMC5592248 28689664

[29] Bamberger EE, Ben-Shimol S, Abu Raya B, Katz A, Givon-Lavi N, Dagan R, et al. Pediatric invasive Haemophilus influenzae infections in Israel in the era of Haemophilus influenzae type b vaccine: a nationwide prospective study. Pediatr Infect Dis J. 2014;33(5):477–81. 10.1097/INF.0000000000000193 24445822

[30] Weinberger DM, Klugman KP, Steiner CA, Simonsen L, Viboud C. Association between respiratory syncytial virus activity and pneumococcal disease in infants: a time series analysis of US hospitalization data. PLoS Med. 2015;12(1):e1001776. 10.1371/journal.pmed.1001776 PMC4285401 25562317

