# Supplementary material for: Integrated host–microbe biomarkers for early diagnostic classification of paediatric LRTI in mechanically ventilated children
Source: Respir Res. 2026 Jul 27;27:425. doi: 10.1186/s12931-026-03829-6 (PMC13721528; doi:10.1186/s12931-026-03829-6)

SF1. ***Flow chart of the analytical framework utilised in this study.***

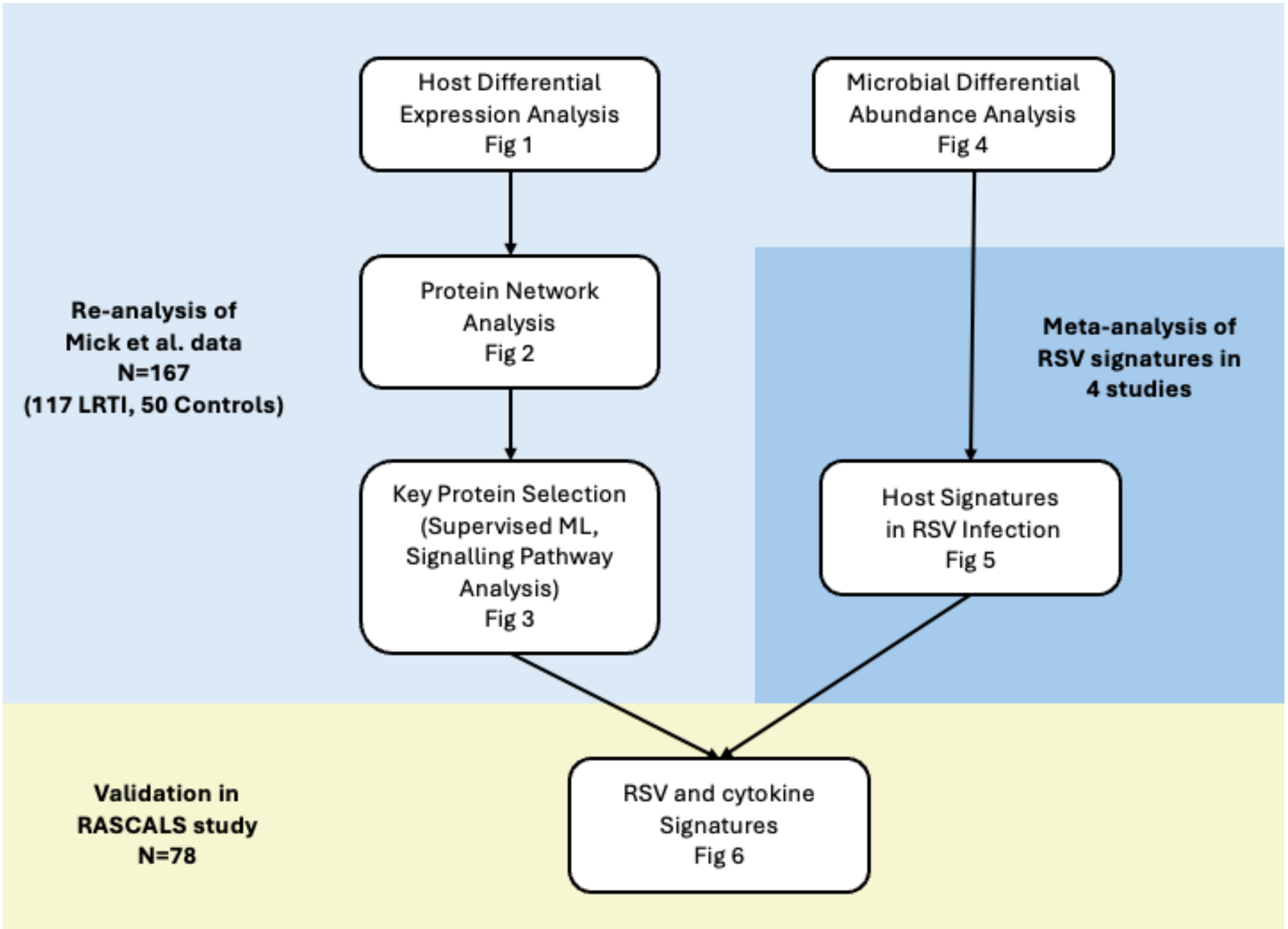

SF2. Plot showing all nodes and edges in the full LRTI network including input proteins and all hidden layer proteins. Nodes with larger sizes have higher eigenvector centrality. The node colour reflects the $\log_2$ fold change in gene expression, with red indicating positive values, blue indicating negative values, and white indicating near-zero or N/A values.

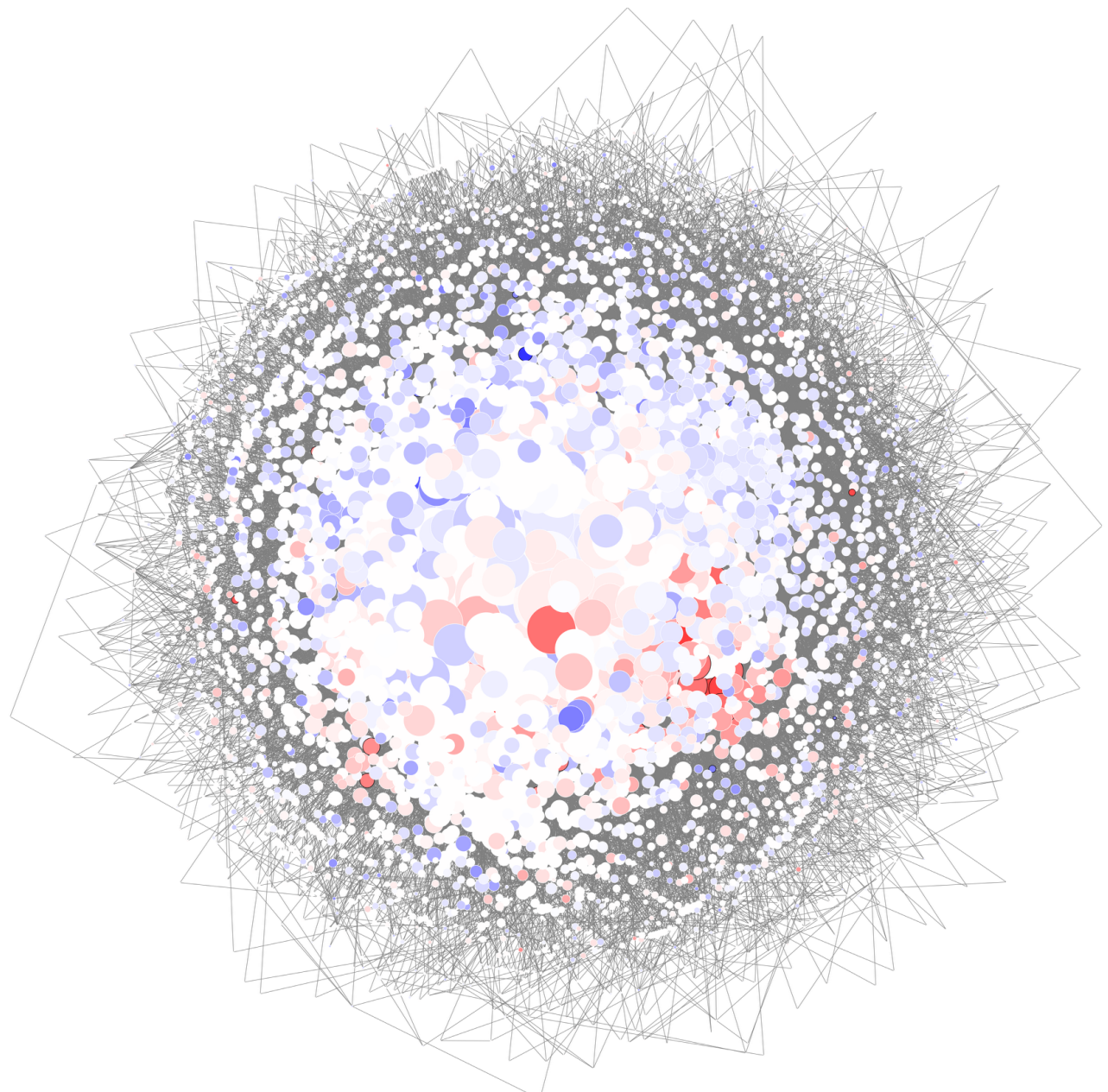

SF3. Plot showing the subnetwork which includes all edges containing key or input proteins. Nodes with larger sizes have higher eigenvector centrality. The node colour reflects the $\log_2$ fold change in gene expression, with red indicating positive values, blue indicating negative values, and white indicating near-zero or N/A values.

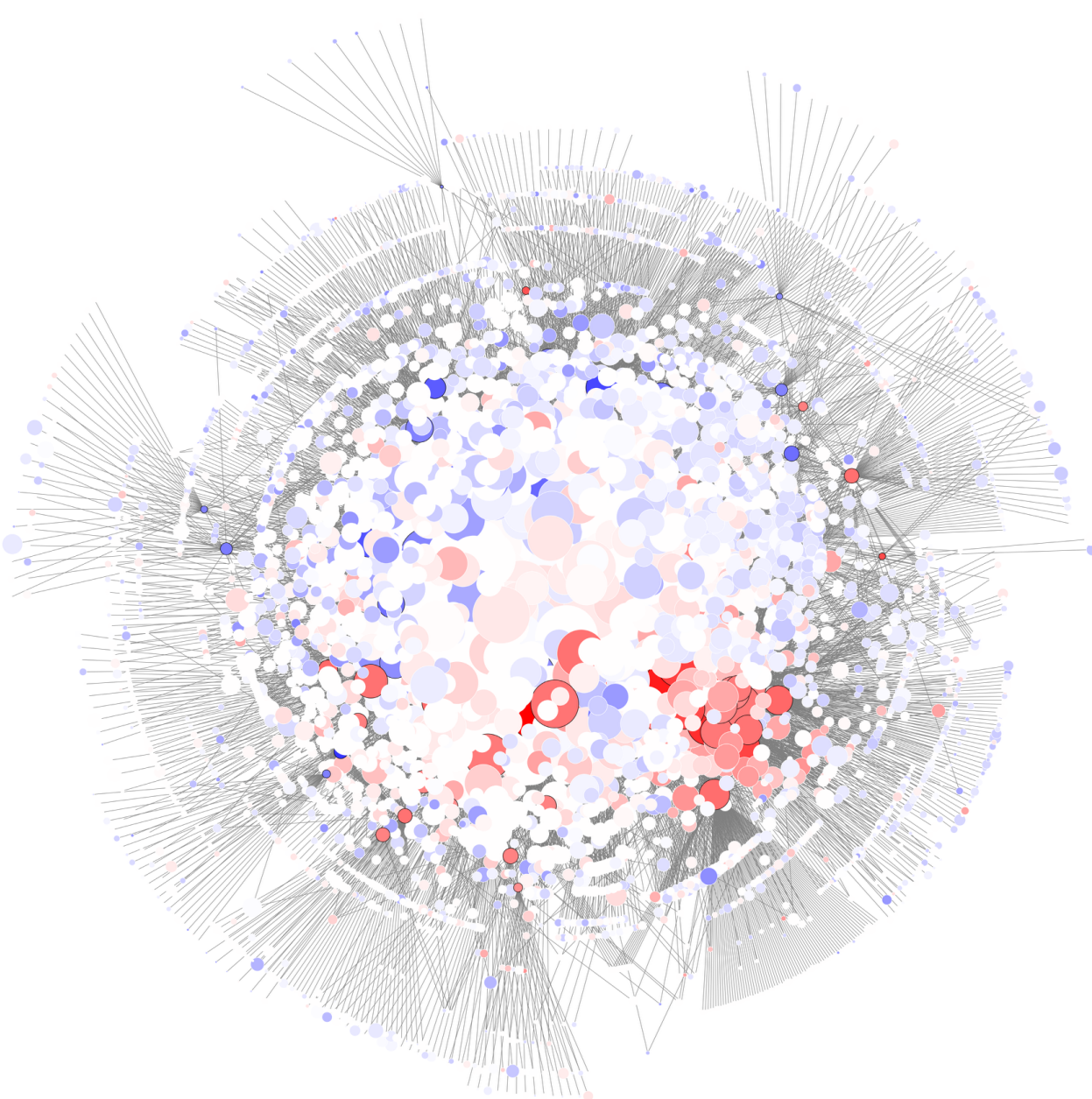

SF4. All LR classifiers using key genes in each pathway. Pathways in descending order of the mean F1 score across 5 folds.

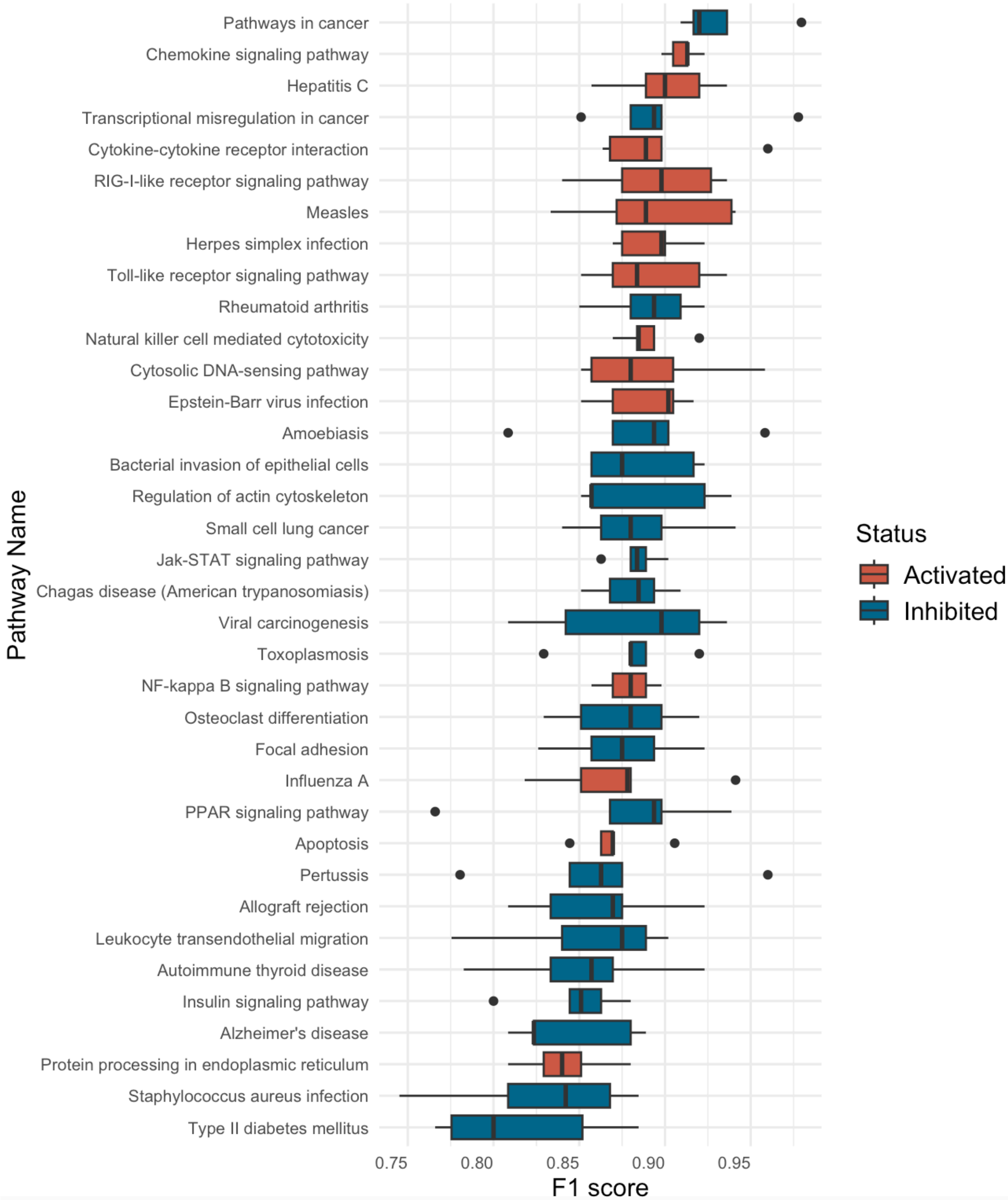

SF5. Volcano plot on bacteria/fungi grouped by genus using DESeq2.

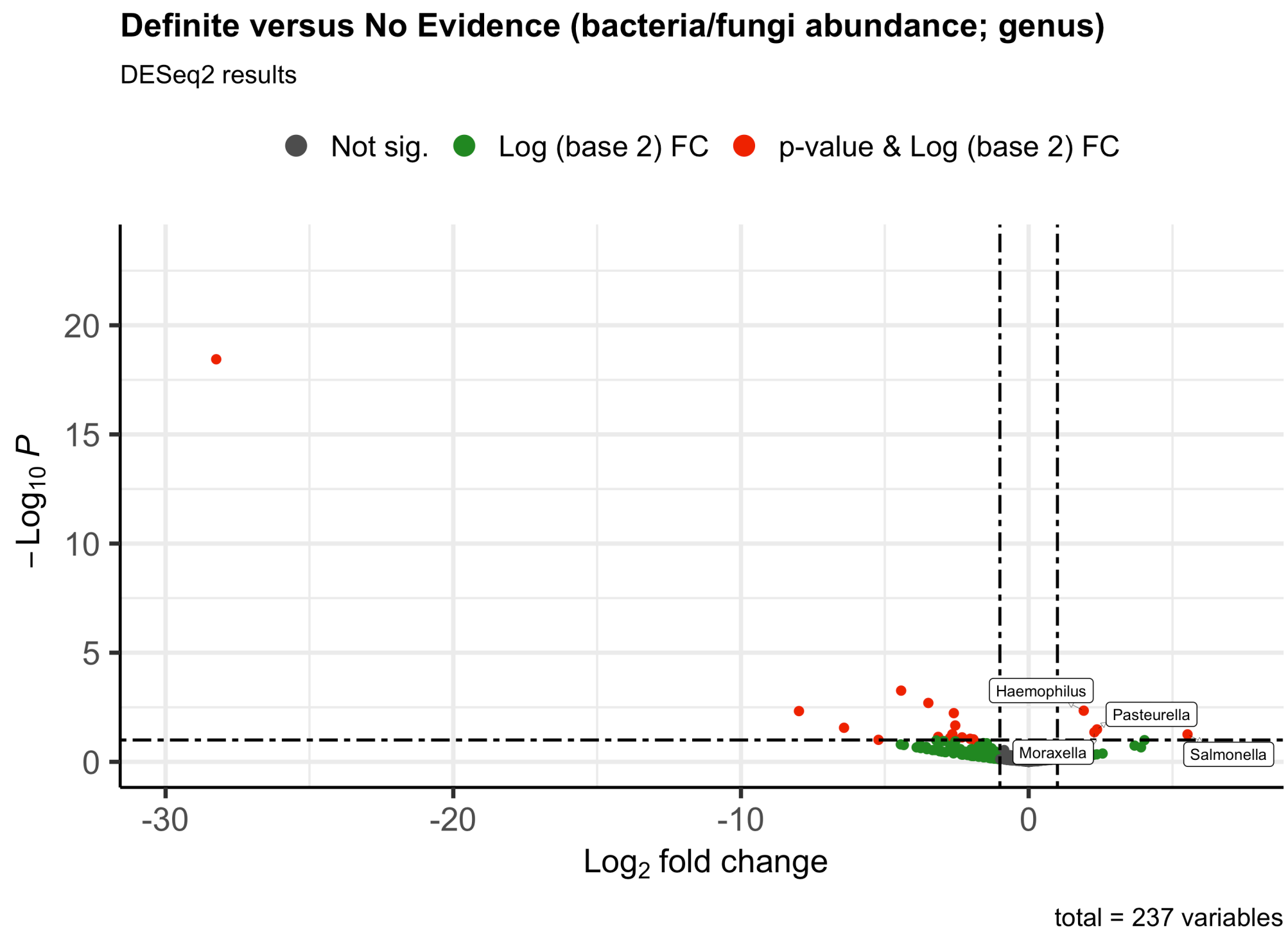

SF6. Volcano plot on bacteria/fungi grouped by genus using edgeR.

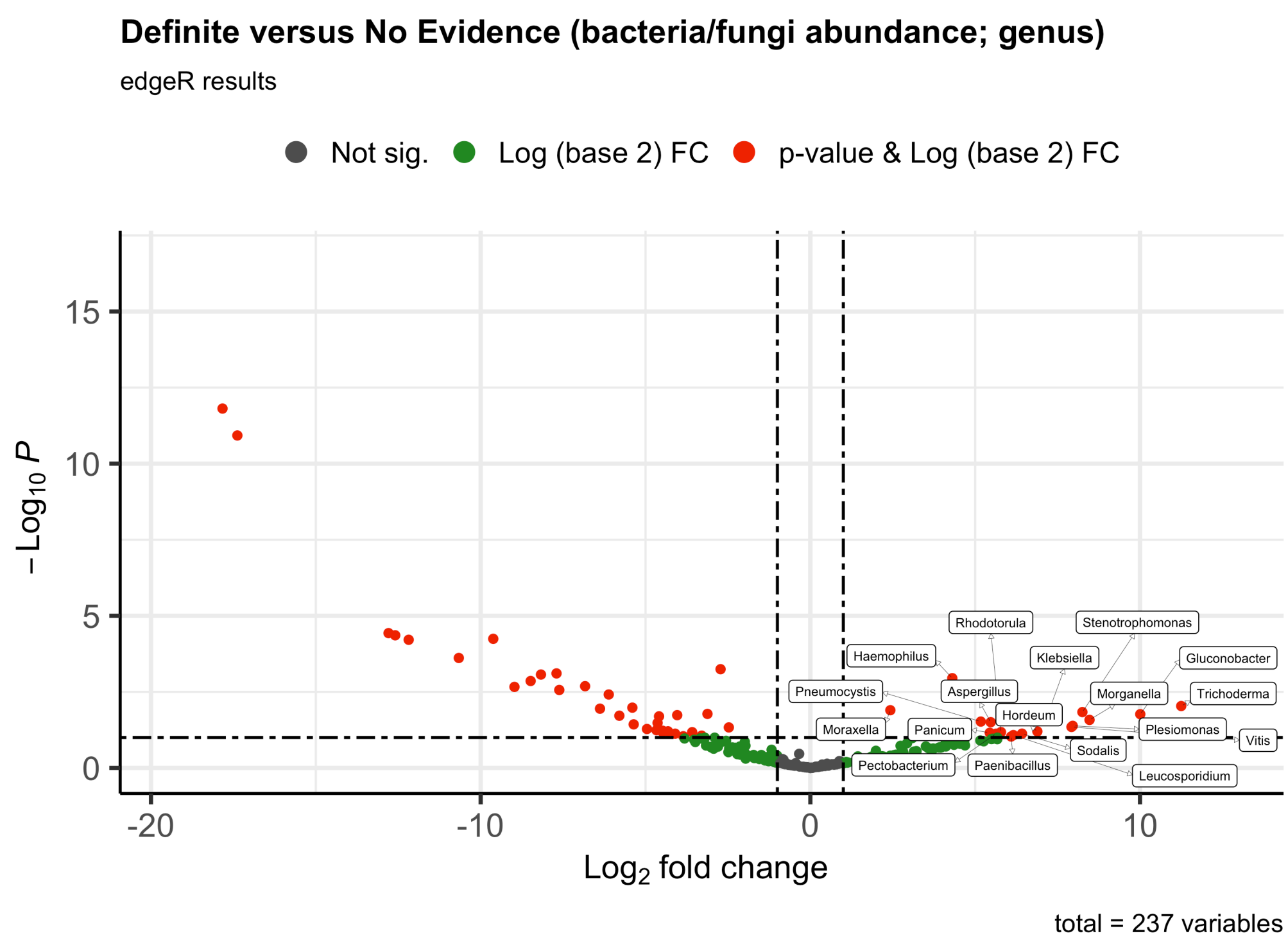

SF7. Volcano plot on bacteria/fungi grouped by species using DESeq2.

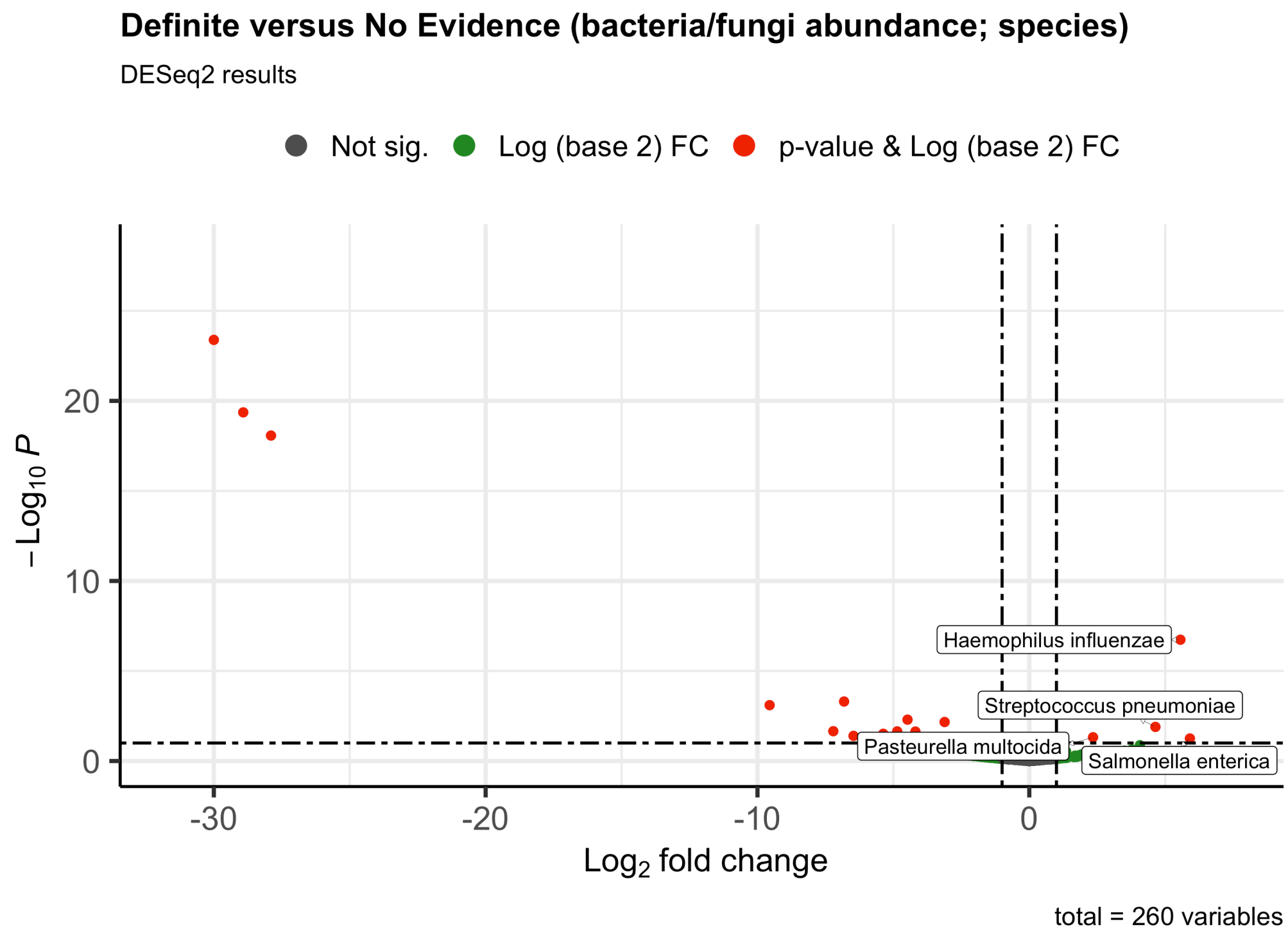

SF8. Volcano plot on bacteria/fungi grouped by species using edgeR.

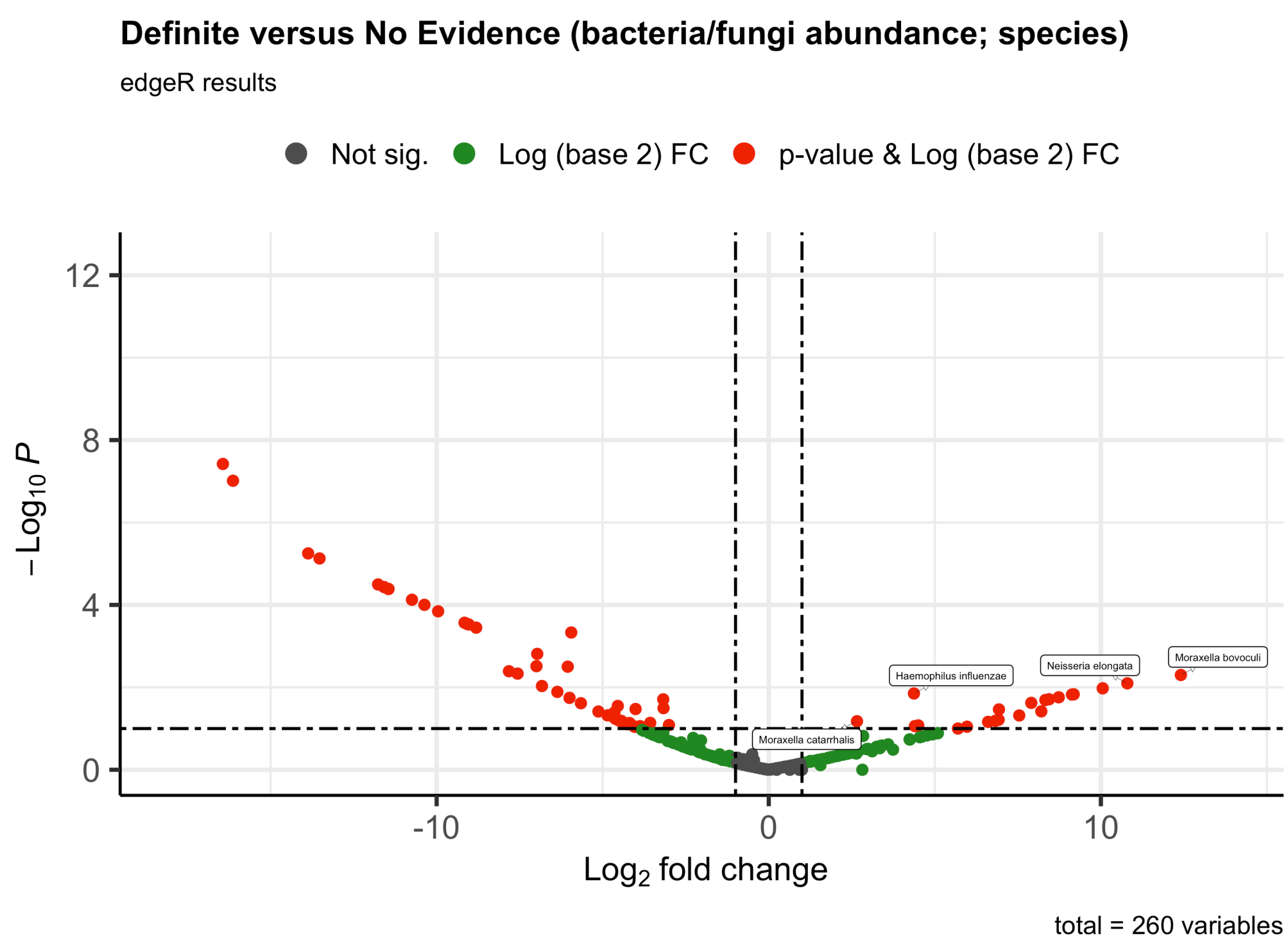

SF9. Plot showing all nodes and edges in the full RSV-host network including input, output, and all hidden layer proteins. Nodes with larger sizes have higher eigenvector centrality. The node colour reflects the log_2 fold change in gene expression, with red indicating positive values, blue indicating negative values, and white indicating near-zero or N/A values.

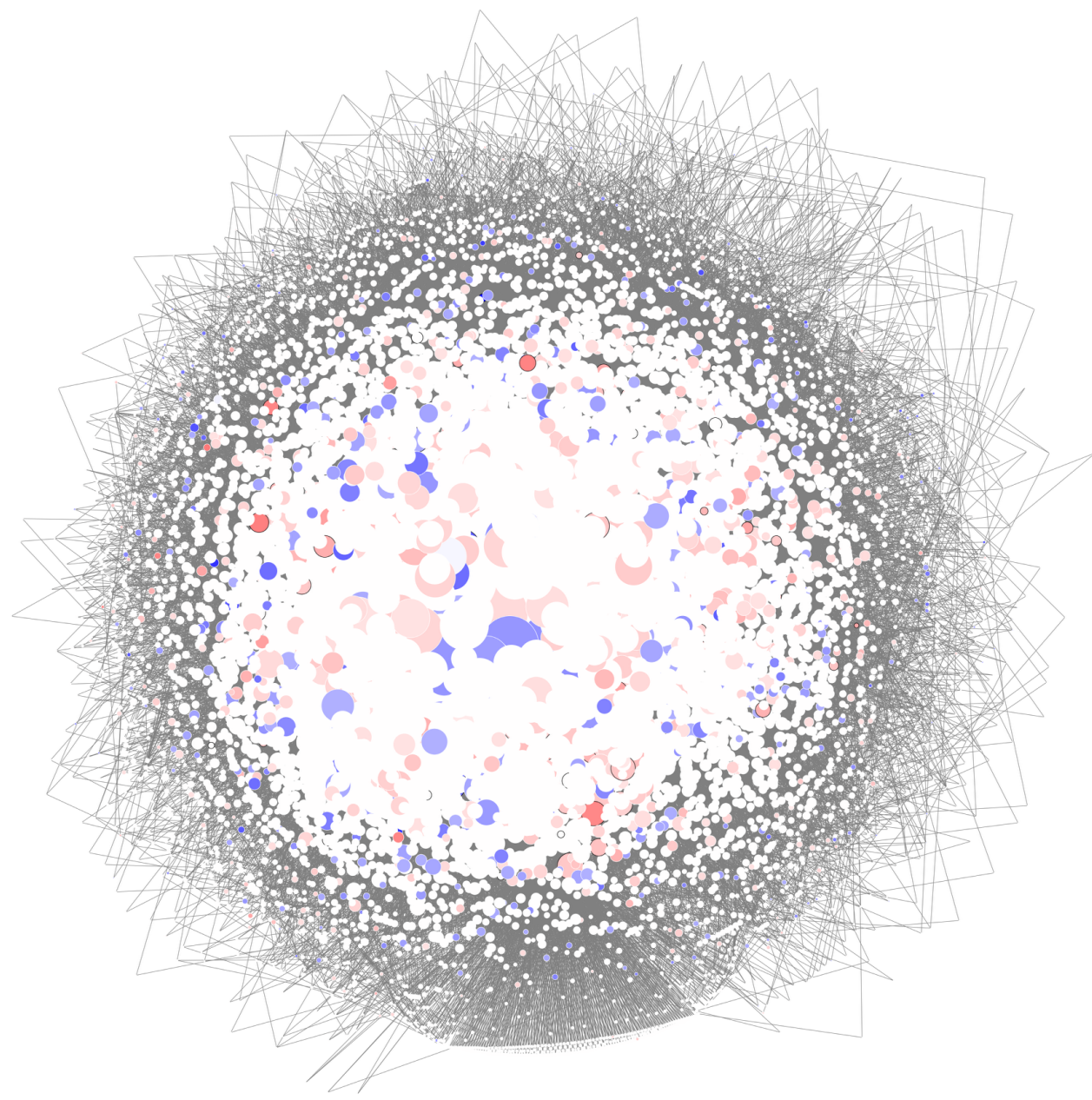

SF10. Plot showing the subnetwork which includes all edges containing key, input and output proteins. Nodes with larger sizes have higher eigenvector centrality. The node colour reflects the log_2 fold change in gene expression, with red indicating positive values, blue indicating negative values, and white indicating near-zero or N/A values.

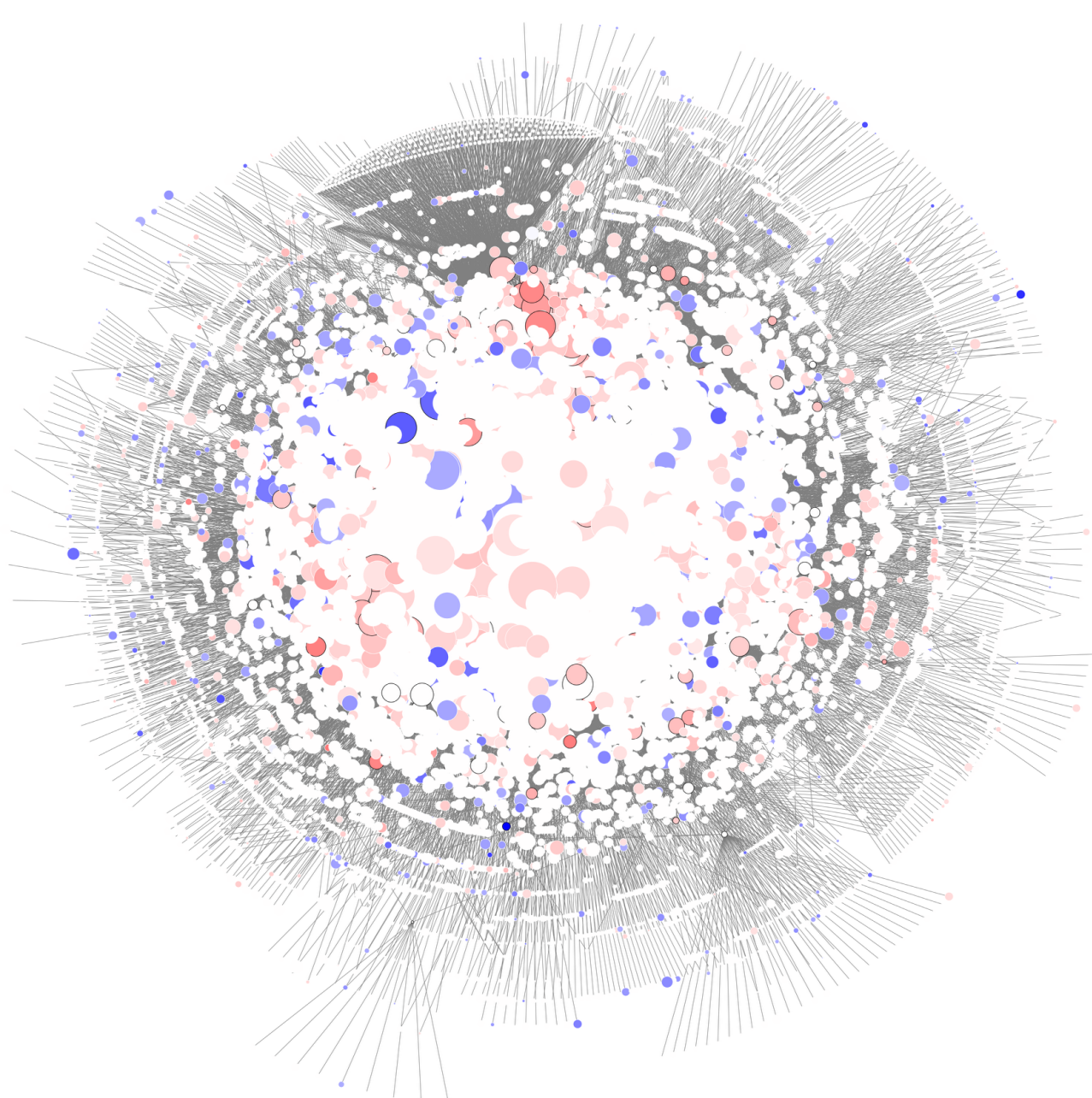

SF11. Graphic illustration of the nested 5-fold cross-validation method. The best-performing model from the inner loop is used to train the train fold in the outer loop.

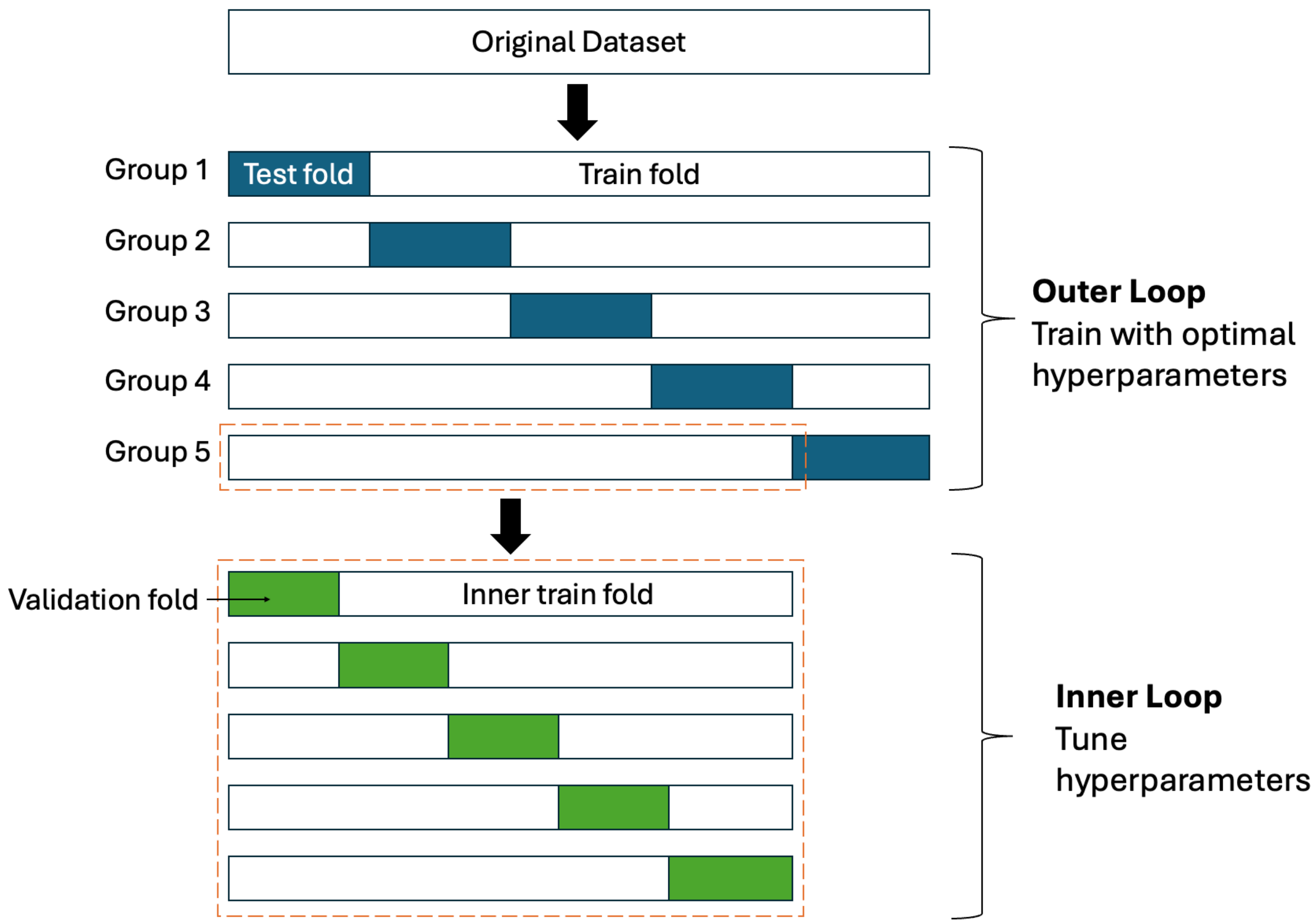

SF12. A) to E): Comparison of mini-BAL level of A) IL-1β, B) IL-4, C) IL-8, D) IL-6, and E) TRAIL between bacterial LRTI and non-bacterial group. F) to J): Comparison of plasma blood level of F) IL-1β, G) IL-4, H) IL-8, and I) IL-6, and J) TRAIL between bacterial LRTI and non-bacterial group.

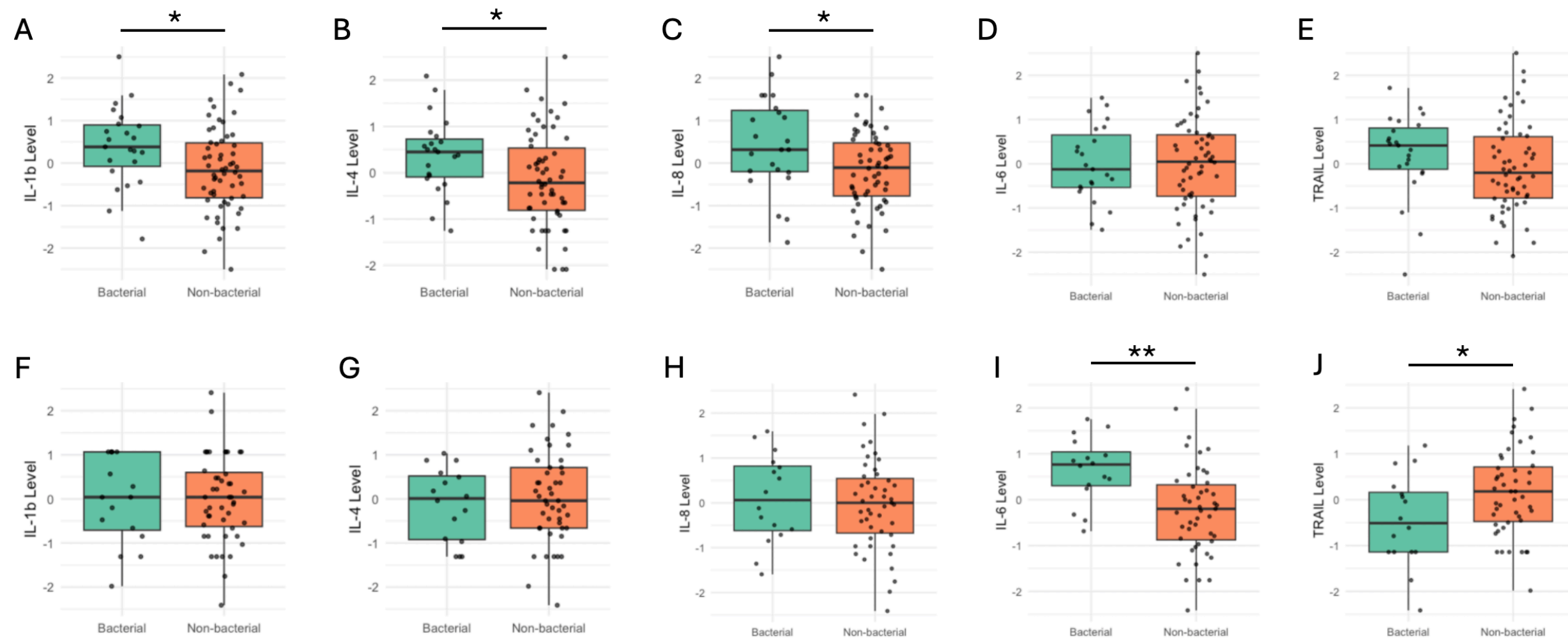

SF13. A) to C): Comparison of mini-BAL level of A) IL-1β, B) IL-4, and C) IL-8 among bacterial LRTI, non-bacterial LRTI, and non LRTI groups. D) to E): Comparison of plasma blood level of D) IL-6 and E) TRAIL among bacterial LRTI, non-bacterial LRTI, and non LRTI groups.

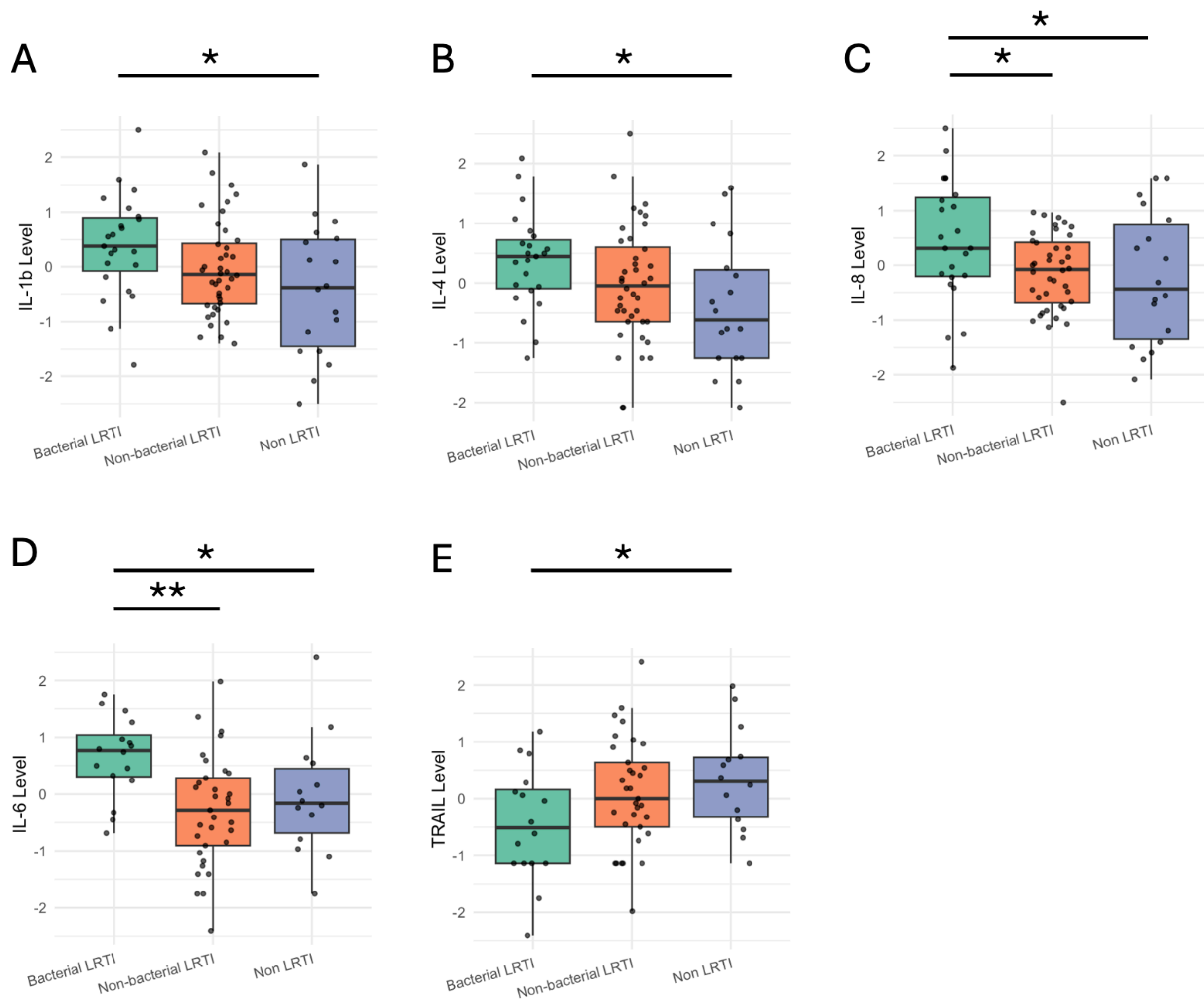

Supplement: Supplementary file 1 [file 12931_2026_3829_MOESM2_ESM.pdf]
